# Zinc alleviates stroke development through autophagy-mediated modulation of immune microenvironment

**DOI:** 10.3389/fimmu.2025.1666225

**Published:** 2025-09-03

**Authors:** Zijie Qiu, Xin Liu, Jie Li, Hongming Yin, Jiankun Luo, Zigang Lin, Mayire Rexiati, Adila Abulaiti, Aikebaier Yasen, Lifei Xing, Dilihumaer Aili, Bing Meng, Xiaoqi Li, Zhenhua You, Jiaxin He, Chaowen Huang, Zhenzhu Qian, Jialong Chen

**Affiliations:** ^1^ Dongguan Key Laboratory of Environmental Medicine, School of Public Health, Guangdong Medical University, Dongguan, China; ^2^ Kashi University School of Medicine, Kashi, Xinjiang, China; ^3^ Department of Neurology, Dongguan Institute of Respiratory Medicine, The First Dongguan Affiliated Hospital of Guangdong Medical University, Dongguan, China; ^4^ Department of Neurology, Dongguan Shipai Hospital Dongguan, Dongguan, China; ^5^ Department of Pulmonary and Critical Care Medicine, Jiangmen Institute of Respiratory Disease, Jiangmen Central Hospital, Jiangmen, China

**Keywords:** stroke, neuroinflammation, zinc, autophagy, immune cell, micronutrients

## Abstract

**Background:**

Stroke is a leading cause of death and long-term disability worldwide and is increasingly recognized as a neuroinflammatory, immune-mediated disease. Acute ischemia triggers robust activation and infiltration of innate immune cells, exacerbating neuronal injury. Zinc is an essential micronutrient with known immunomodulatory and autophagy-regulating roles, but its impact on stroke-driven neuroinflammation remains unclear. We aimed to investigate whether zinc protects against ischemic brain injury by modulating autophagy and the immune microenvironment.

**Methods:**

Genome-wide association (GWAS) and NHANES cohort analyses were performed to assess the association between zinc intake levels and stroke risk. Pathway enrichment analysis, PPI network construction, and diagnostic modeling were undertaken to identify ZIARs (zinc-responsive immuno-autophagic regulators). Immune infiltration analysis was used to assess immune cell infiltration levels in stroke. Cell-based ischemia-reperfusion experiments were conducted to evaluate zinc’s effects on autophagy and neuronal cell injury.

**Results:**

Higher zinc levels were associated with lower stroke prevalence, as supported by GWAS results (OR ~0.141) and NHANES findings (adjusted OR ~0.96). Zinc-associated genes were predominantly enriched in autophagy and inflammatory signaling pathways, including PI3K-Akt and NF-κB. The diagnostic model identified ZIARs whose expression was closely linked to immune cell composition, particularly neutrophil infiltration, in stroke. *In vitro* experiment, zinc pretreatment of hypoxia-stressed neurons reduced cell death and oxidative damage, whereas autophagy inhibition abolished zinc’s neuroprotective effect. qPCR and Western blot analyses further confirmed that zinc attenuates RELA-driven autophagy overactivation, thereby promoting neuronal survival.

**Conclusion:**

Zinc confers neuroprotection in ischemic stroke by restoring autophagic flux and suppressing excessive innate inflammation through regulation of ZIARs. These findings underscore zinc’s neuroprotective role via immune-autophagy crosstalk and position it as a potential strategy for stroke prevention and intervention.

## Graphical Abstract

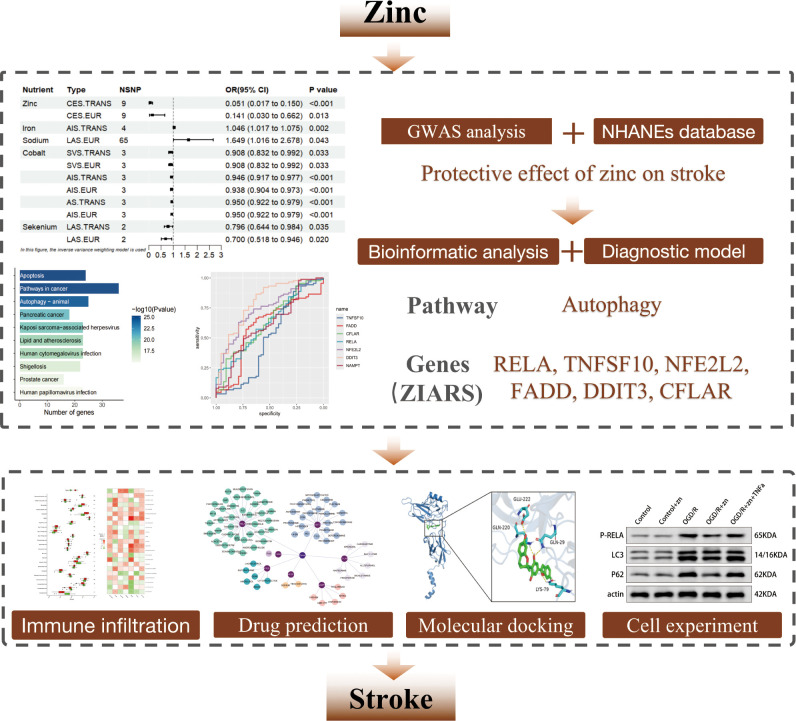

## 1 Introduction

Stroke is characterized by acute cerebral ischemia that triggers neuroinflammatory cascades and immune-mediated secondary brain injury (1). It remains a leading cause of death and long-term disability worldwide (2). Recent epidemiological data from the World Stroke Organization (WSO) indicate a substantial global increase in stroke incidence, mortality, and related disabilities between 1990 and 2021 (3). This rising trend is exacerbated by an aging population and the persistence of modifiable risk factors, including smoking, alcohol consumption, physical inactivity, and nutritional deficiencies (4, 5).

Accumulating evidence underscores that inflammation and immune activation are pivotal contributors to the pathogenesis of stroke. Following cerebral ischemia, the robust activation of innate immune cells, such as neutrophils, macrophages and microglia, initiates inflammatory responses characterized by elevated levels of pro-inflammatory cytokines, oxidative stress, and disruption of the blood-brain barrier (BBB) (6, 7). These immune-mediated events further amplify neuronal injury, contributing significantly to post-stroke pathology and clinical outcomes. Consequently, understanding and modulating the inflammatory and immune mechanisms in stroke has emerged as a promising therapeutic strategy.

In this context, micronutrients have attracted increasing attention due to their immunomodulatory and anti-inflammatory properties. Among various micronutrients, zinc is particularly notable for its integral role in regulating immune function, autophagic flux, and oxidative stress responses (8, 9). Previous studies have indicated that zinc deficiency exacerbates immune dysfunction and inflammatory processes, while adequate zinc levels have been associated with improved immune homeostasis and reduced inflammatory responses (10, 11).

Nevertheless, the direct relationship between zinc intake levels and stroke-associated neuroinflammation remains poorly understood. Clarifying this relationship could provide novel insights into stroke pathophysiology and potential therapeutic avenues. Therefore, our study used the NHANES database and GWAS analysis to examine correlations between micronutrient intake (particularly zinc intake level) and stroke. Subsequently, pathway enrichment, PPI network, diagnostic models and immune infiltration analysis were employed to identify the core targets and functional pathways linking zinc to stroke, emphasizing autophagy-related mechanisms. Cell-based experiments further corroborated the role of zinc in the pathogenesis of stroke. We found that zinc restrains excessive autophagy via RELA inhibition, a neuroprotective mechanism validated by qPCR and Western blot. Building on this mechanistic insight, potential therapeutic compounds targeting RELA emerged as candidates for stroke treatment. This study provides a comprehensive evaluation of zinc’s therapeutic potential in stroke, offering a theoretical basis for zinc-based strategies in stroke prevention and therapy.

## 2 Materials and methods

### 2.1 Bioinformatic methods

#### 2.1.1 GWAS analysis

This study employed a GWAS data to investigate causal relationships linking micronutrients to several stroke phenotypes using single nucleotide polymorphisms (SNPs) as instrumental variables (IVs). Genetic associations with micronutrients were obtained from the largest European GWAS (12–15), identifying SNPs for each micronutrient at *p*< 5 × 10^−6^. Thirteen micronutrients were included: zinc, calcium, iron, copper, molybdenum, selenium, chromium, sodium, magnesium, cobalt, phosphorus, vitamin A, and vitamin C. For stroke, data for Atherosclerotic Stroke (AS), Acute Ischemic Stroke (AIS), Large Artery Atherosclerosis Stroke (LAS), Cardioembolic Stroke (CES), and Small Vessel Stroke (SVS) were obtained from the MEGASTROKE consortium (EUR n ≈ 446,696; trans-ancestry n ≈ 521,612). Independence was enforced by LD clumping using PLINK (–clump, r² < 0.001, window = 10,000 kb) against the 1000G-EUR reference panel. Causal effect estimates were derived using inverse-variance weighted (IVW) meta-analysis of SNP-specific Wald ratios, with sensitivity analyses performed using MR-Egger, weighted median, weighted mode, and simple mode estimators (16, 17). Horizontal pleiotropy and outlier detection were assessed using MRPRESSO. Instrument strength (F-statistic > 10), pleiotropy (MR-Egger intercept *p* > 0.05), and heterogeneity (Cochran’s Q) were assessed (18–20). All analyses were conducted using R version 4.3.0.

#### 2.1.2 NHANES database analysis

We analyzed data from the U.S. National Health and Nutrition Examination Survey (NHANES) 2009 – 2018 (n = 47,652) to examine the association between dietary zinc intake and stroke prevalence (https://www.cdc.gov/nchs/). After excluding individuals with missing covariates, 9,655 participants aged 18 – 79 were included (9,293 non-stroke; 362 with stroke), and all participants provided informed consent. Dietary zinc intake (mg/day) was assessed by averaging two 24-hour dietary recalls. Stroke history was defined by self-report of ever being told by a doctor of a stroke. Extreme zinc intake outliers were excluded using the interquartile range (IQR) method, in which the lower bound was defined as Q1 – 1.5 × IQR and the upper bound as Q3 + 1.5 × IQR; values outside these bounds were considered outliers. Statistical analyses were performed using R software (version 4.3.0) and EmpowerStats 2.2. NHANES sampling weights were applied to compare baseline characteristics between the stroke and non-stroke groups. Weighted logistic regression models assessed the association between zinc intake and stroke risk, with three models used: unadjusted; model 1 adjusted for age, sex, and race; and model 2 adjusted for age, sex, race, income, education, smoking status, BMI, hypertension, diabetes/fasting glucose, estimated glomerular filtration rate (eGFR), and triglycerides. Subgroup analyses were conducted by key covariates. A generalized additive model (GAM) was used to explore dose–response relationships between zinc intake and stroke risk. A two-tailed p-value < 0.05 was considered statistically significant.

#### 2.1.3 Identification of zinc-autophagy-related targets in stroke

We retrieved zinc-related and stroke-related targets from the GeneCards, DrugBank, and OMIM databases, using “Homo sapiens” species. We filtered targets to include only those with a GeneCards relevance score ≥ the median and removed duplicates. Using R (version 4.3.0), we then identified the overlapping targets between the zinc-related and stroke-related lists. Because autophagy appeared to be involved in stroke pathology, we retrieved autophagy-related targets from the HADb database and intersected them with the overlapping list to pinpoint zinc–autophagy–stroke targets.

#### 2.1.4 Construction of protein-protein interaction network and MCODE analysis

The targets identified in section 2.1.3 were imported into the STRING database for protein-protein interaction analysis. The network was visualized using Cytoscape 3.8.0, and clustering was performed with the MCODE plugin using degree cutoff ≥ 2 and k-core ≥ 2. MCODE plugin identified three groups of PPI network targets: (i) potential targets of zinc-autophagy related to disease, (ii) modular network 1 targets based on MCODE analysis, and (iii) modular network 2 targets based on MCODE analysis.

#### 2.1.5 Functional enrichment of the targets

Targets from section 2.1.4, including potential targets, modular network 1 targets, and modular network 2 targets, were analyzed in the DAVID database for GO and KEGG pathway enrichment (21), using “Homo sapiens” species (*p* < 0.05). This study concentrated on GO-BP and KEGG analysis results, which provide insights into cellular functions and regulatory pathway processes.

#### 2.1.6 Construction of a diagnostic model for ZIARs

We integrated transcriptomic datasets from the Gene Expression Omnibus (GEO; https://www.ncbi.nlm.nih.gov/geo/). Two independent cohorts were included: GSE22255 (20 ischemic stroke patients and 20 controls) and GSE58294 (69 patients and 23 controls). Both were generated on the Affymetrix Human Genome U133 Plus 2.0 platform (GPL570), which offers genome-wide coverage of protein-coding transcripts. Patient samples were derived from peripheral blood mononuclear cells (PBMCs), a recognized proxy for systemic immune responses after cerebral ischemia (22, 23). Expression data were corrected for batch effects with sva and normalized with limma to ensure cross-cohort comparability.

For machine learning modeling, we used the genes identified in Section 2.1.4 as input features without additional filtering. Data were split into training (70%) and testing (30%) sets with a fixed random seed (123). Within the training set, 5-fold cross-validation repeated three times was applied for hyperparameter tuning. Four models were constructed: (i) Random Forest (RF) with 500 trees and mtry ∈ {2, √p, p/3}; (ii) Support Vector Machine (SVM) with a radial kernel (σ from sigest) and C ∈ {0.25, 0.5, 1}; (iii) Extreme Gradient Boosting (XGBoost, xgbDART) with nrounds ∈ {50, 100, 150}, max_depth ∈ {3, 6, 9}, eta ∈ {0.3, 0.1, 0.01}, gamma ∈ {0, 0.1}, and colsample_bytree = subsample = 0.8; (iv) Generalized Linear Model (GLM) with logistic regression (no tuning).

Model selection was based on AUC maximization under cross-validation. Interpretability was assessed with DALEX, which ranked feature importance and identified the top five discriminative genes, termed zinc-responsive immuno-autophagic regulators (ZIARs). Performance was further evaluated by residual diagnostics and ROC analysis (pROC), ensuring both predictive accuracy and biological interpretability.

#### 2.1.7 Immune cell infiltration and correlation analysis

Immune alterations accompanying stroke were quantified on PBMC-derived transcriptomes (GSE22255 and GSE58294) using single-sample gene set enrichment analysis (ssGSEA) as implemented in GSVA, yielding sample-wise enrichment scores (24). Immune cell marker sets were taken from Charoentong et al (25)., which compile lineage-level leukocyte signatures (e.g., neutrophils, monocytes/macrophages, NK cells, dendritic cells, B-/T-cell subsets) and have been broadly reused for peripheral blood deconvolution across diseases; in the present PBMC context we therefore interpret scores as peripheral leukocyte programs, not CNS-resident glia (25). Group differences between patients and controls were evaluated by Wilcoxon rank-sum tests, and Spearman correlations were computed between ZIARs and immune scores; results were visualized as boxplots and correlation heatmaps. Boxplots show the median and interquartile range (IQR) with whiskers extending to 1.5×IQR. P values for the 22 cell-type comparisons and for ZIAR–cell correlations were two-sided and Benjamini–Hochberg FDR–adjusted. This PBMC-focused approach aligns with the well-recognized contribution of systemic immune responses to stroke pathophysiology (26, 27).

#### 2.1.8 Drug prediction and molecular docking

Drug data for ZIARs were retrieved from the Drug-Gene Interaction Database (DGIdb) (Cotto et al., 2018), and drug-gene interactions were visualized using Cytoscape. ZIARs were uploaded to the STRING database to construct a PPI network. Molecular docking was performed with the top 4 drugs predicted for these hub genes based on interaction score. The 3D structures of drugs were downloaded from the PubChem database (https://www.ncbi.nlm.nih.gov/pubmed/), converted into mol2 format using OpenBabel software, and processed with Autodock Tools (28) for hydrogenation and rotatable bond setting before being saved in PDBQT format. The PDB structure files of target proteins were downloaded from the RCSB Protein Data Bank (https://www.rcsb.org/search), water molecules were removed, hydrogen atoms were added, and the files were stored in PDBQT format. Molecular docking was performed using Autodock Vina 1.2.5, and the results were visualized with Pymol 2.5.5 and Discovery Studio (29).

### 2.2 Cell experiment validation

#### 2.2.1 Cell culture

HT-22 mouse hippocampal neurons (ATCC, HTA2020) were cultured in high-glucose DMEM (Gibco, C11995500BT) with 10% FBS (FBS, Gibco, 10270 - 106) and 1% penicillin/streptomycin (HyClone, SV30010) at 37 °C, 5% CO^2^, and 95% humidity. Cells were subcultured every 2 – 3 days with 0.25% trypsin-EDTA (Gibco, 25200056) and used in the logarithmic growth phase.

#### 2.2.2 Construction of experimental grouping

To construct the oxygen-glucose deprivation/reoxygenation (OGD/R) model, cells (1×10^4^ per well) were seeded in 96-well plates and cultured for 24 hours, after which the medium was replaced with glucose-free DMEM (Gibco, 11966025). Cells were subjected to hypoxia (94% N^2^, 5% CO^2^, 1% O^2^) at 37 °C for 6 hours (30). After reoxygenation (21% O^2^) for 24 hours, reperfusion injury was simulated. Four groups were tested:(1) Control, normoxia; (2) Zinc, 50 uM ZnCl^2^ 2h under normoxia; (3) OGD/R, 6 h hypoxia (1% O^2^, glucose-free DMEM), followed by 24h reoxygenation; (4) OGD/R + zinc, 2h ZnCl^2^ pretreatment, with zinc maintained throughout OGD/R; To assess RELA involvement, 10 ng/ml TNF-α was added during hypoxia in the OGD/R + Zn group to mimic early inflammatory signaling; fresh zinc-containing medium was used during reoxygenation. TNF-α was freshly diluted from endotoxin-free stock (LAL assay <0.01 EU/μg; Lonza #50-647U). Vehicle controls (PBS + 0.1% BSA) were included in all OGD/R groups.

#### 2.2.3 Cell viability analysis of CQ-treated comparison models

To assess autophagy’s role in zinc-mediated neuroprotection, we added an OGD/R + zinc+ CQ group. In this group, cells were pre-treated with ZnCl^2^ for 2 h, then subjected to OGD/R in the presence of 10 μM chloroquine (CQ, Sigma, C6628) added at the start of reoxygenation and maintained for 24 h (31). Cell viability was assessed using the CCK - 8 kit (Dojindo, CK04), 10 μL of CCK - 8 solution was added to each well, incubated at 37 °C in the dark for 2 hours, with absorbance measured at 450 nm. Data were normalized to the blank control group (100%).

#### 2.2.4 qPCR analysis

Total RNA was extracted using TRIzol reagent, treated with DNase I to remove genomic DNA, and reverse-transcribed with the PrimeScript™ RT kit. qPCR was performed on a Bio-Rad system using SYBR Green reagent, with GAPDH as the internal control (forward:5′-CATCACTGCCACCCAGAAGACTG-3′, reverse: 5′-ATGCCAGTGAGCTTCCCGTTTCAG-3′, 153 bp) and RELA-specific primers (forward: 5′-TCCTGTTCGAGTCTCCATGCAG-3′, reverse: 5′-GGTCTCATAGGTCCTTTTGCGC-3′, 150 bp). Relative expression was calculated using the 2^−ΔΔCt method. Data are presented as mean ± SEM and analyzed by one-way ANOVA with Tukey’s *post hoc* test.

#### 2.2.5 Western blot analysis

Cells were lysed with RIPA buffer (Beyotime, P0013B) containing protease inhibitors for 30 minutes. Lysates were centrifuged at 12,000×g for 15 minutes at 4 °C. Protein concentration was measured using the BCA method (Thermo, 23225), and 30 μg protein was separated by 12% SDS-PAGE and transferred to PVDF membrane (Millipore, IPFL00010). After blocking with 5% milk, membranes were incubated with primary antibodies: p-RELA (Ser536) (1:1000, CST, 3033), LC3 (1:1000, CST, 4108), p62 (1:1000, Abcam, ab56416) and β-actin (1:5000, Proteintech, 66009 - 1-Ig) overnight at 4 °C. LC3 and P62 are common autophagy markers (32). Secondary antibody (HRP-conjugated goat anti-rabbit/mouse IgG, 1:5000, Proteintech) was incubated at room temperature for 1 hour. After chemiluminescent detection (Millipore, WBKLS0500), signals were captured and analyzed using Image Lab 6.1.

#### 2.2.6 Oxidative stress and cell damage assessment

Malondialdehyde (MDA) levels were measured using the thiobarbituric acid method (Beyotime, S0131). After reaction with TBA reagent (95 °C for 60 minutes), absorbance at 532 nm was measured, and MDA concentration was calculated using a malondialdehyde standard curve. LDH release was measured by reacting the supernatant with detection solution and protecting from light for 30 minutes (Beyotime, C0017). Absorbance was measured at 490 nm. The LDH release rate was calculated based on the complete cell lysis group.

#### 2.2.7 Serum concentration measurements

Patient serum samples were collected with ethical approval (ECoJmCH [2025] No.68A), centrifuged at 3000×g for 15 minutes at 4°C, and stored at -80°C. zinc concentration was measured using ICP-MS (ICP-MS, Agilent 7900) with three repetitions for average calculation.

#### 2.2.8 Statistical analysis

Data were expressed as mean ± SD and analyzed using SPSS 23.0 software. One-way ANOVA was followed by Tukey’s test for group comparisons. Statistical significance was defined as *p < 0.05, **p < 0.01, ***p < 0.001. Graphs were plotted using GraphPad Prism 8.0.2.

## 3 Results

### 3.1 Increased zinc intake reduces stroke risk

**Figure 1:**
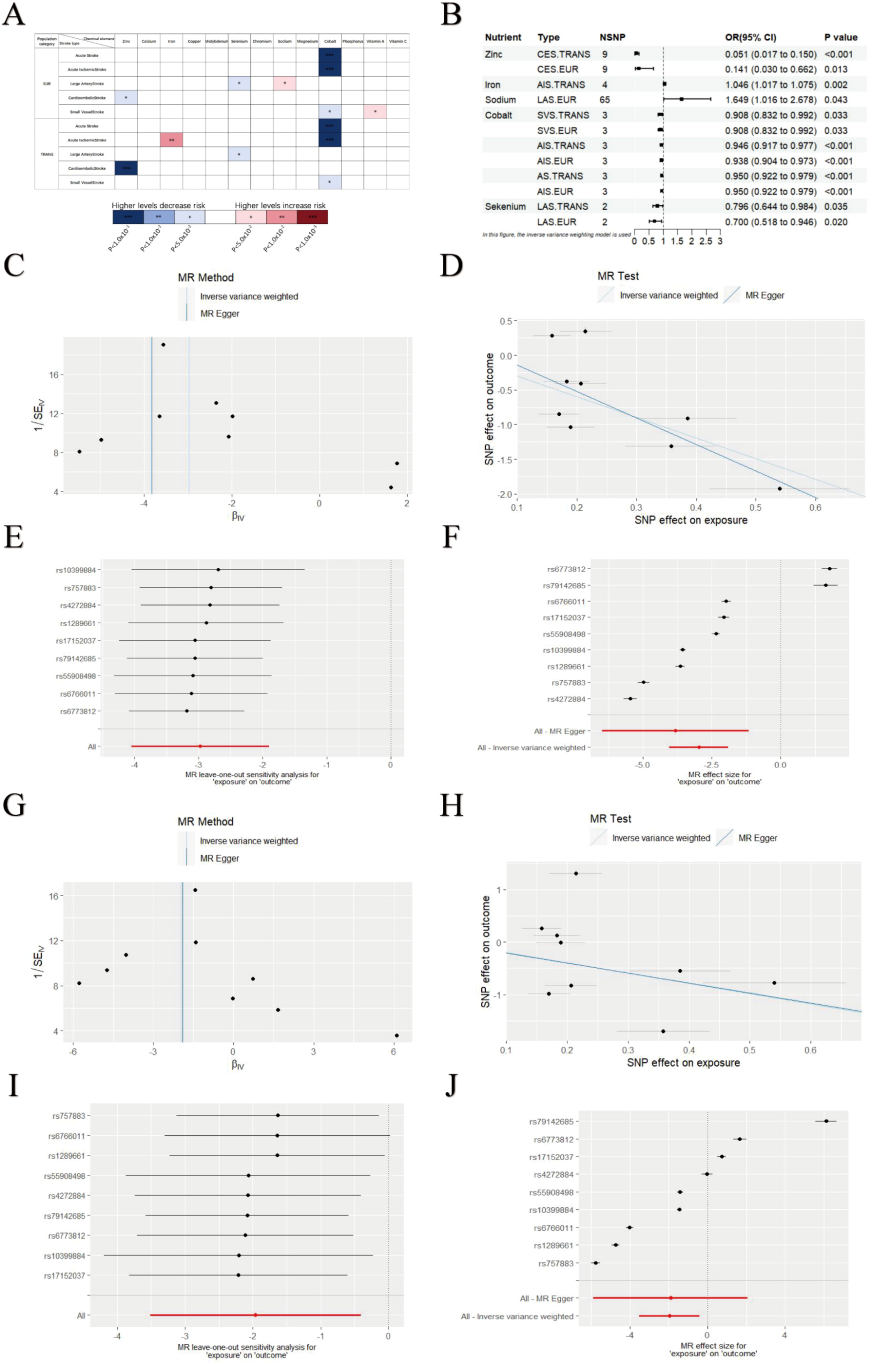
GWAS analysis results of micronutrients levels and stroke. **(A)** GWAS results for various micronutrient intake levels and stroke risk. **(B)** Significant associations between micronutrient levels and stroke. **(C–F)** Leave-One-Out plot, Funnel plot, Forest plot and Scatter plot results for zinc and CES in EUR populations. **(G–J)** Leave-One-Out plot, Funnel plot, Forest plot and Scatter plot results for zinc and CES in TRANS populations. Asterisks = statistical significance: * P ≤ 0.05; ** P ≤ 0.01; *** P ≤ 0.001.

Using GWAS analysis approach with the IVW method, we identified statistically significant associations between various micronutrient intake levels and stroke risk, including zinc, iron, selenium, sodium, cobalt and vitamin A (**Figure 1A**). The association with vitamin A was excluded due to an abnormal beta value (β = 11.024, SE = 4.506) that yielded an implausible odds ratio for stroke risk (**Supplementary Table S1**). In the European cohort, higher genetically predicted zinc levels were associated with a lower risk of CES (OR = 0.141, 95% CI = 0.030–0.662, *P* = 0.013). Similarly, selenium levels correlated inversely with LAS risk (OR = 0.700, 95% CI = 0.518–0.946, *P* = 0.020), while elevated cobalt levels were linked to higher risks of AS (OR = 0.950, 95% CI = 0.922–0.979, *P*<0.001), AIS (OR = 0.938, 95% CI = 0.904–0.973, *P*<0.001), and SVS (OR = 0.908, 95% CI = 0.832–0.992, *P* = 0.033). Conversely, increased sodium levels were associated with a higher risk of LAS (OR = 1.649, 95% CI = 1.015–2.678, *P* = 0.043). Across various regional populations, similar associations were observed for Zinc, selenium, and cobalt, while higher iron levels were associated with an increased risk of AIS (OR = 1.045, 95% CI = 1.017–1.075, *P* = 0.002) (**Figure 1B**). We validated these findings with MR-Egger, Weighted Median, Simple Mode, and Weighted Mode methods, which confirmed the results’ reliability (**Supplementary Table S1**). Sensitivity analysis further confirmed the robustness of the GWAS analysis results. MR-Egger testing for pleiotropy revealed no horizontal pleiotropy (all intercept *p* > 0.05), and Cochran’s Q statistic indicated low heterogeneity among the selected SNPs. The GWAS results consistently showed a negative relationship between zinc intake and stroke risk, supporting the hypothesis that higher zinc intake may reduce stroke risk. The leave-one-out analysis showed that the overall effect estimate remained stable after excluding individual SNPs, and the funnel plot was nearly symmetrical, indicating no publication bias. No significant heterogeneity was found in the scatter plot, reinforcing the robustness of the GWAS findings (**Figures 1C–J**). Reverse GWAS analysis showed no significant positive results (**Supplementary Table S1**).

### 3.2 NHANES analysis further validated dietary zinc reduces stroke risk

**Figure 2:**
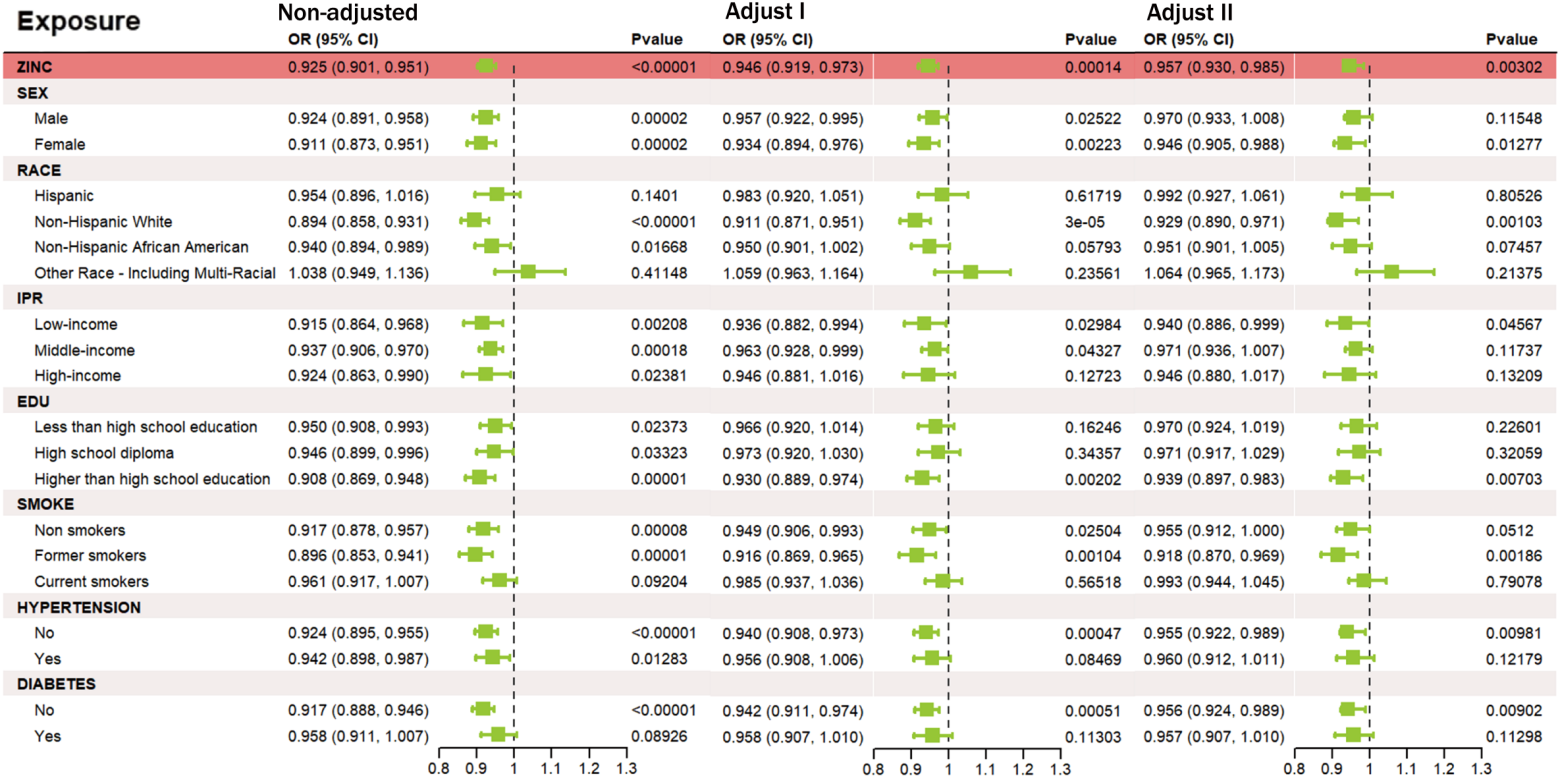
Relationship between zinc intake and stroke risk under different adjustment models. Outcome: stroke; exposure: zinc. (Unadjusted) no covariates; (Model I) age, sex, race; (Model II) age, sex, race, income, education, smoking status, BMI, hypertension, diabetes, triglycerides, and cholesterol.

We analyzed NHANES data from 9,655 participants (aged 18 – 79), of whom 362 had a history of stroke and 9,293 did not (**Figure 2**). The stroke group was older on average (*p* < 0.001), had a higher proportion of non-Hispanic Black individuals (*p* < 0.001), lower education levels (*p* < 0.001), and a higher percentage with low income (p < 0.001) than the non-stroke group. Additionally, the stroke group had higher current smoking rates and a higher prevalence of hypertension and diabetes (p < 0.001). The difference in BMI distribution was not significant (*p* = 0.063). Further details on baseline characteristics are provided in **Table 1**. The association between zinc exposure and stroke risk was assessed using three progressively adjusted models. In the unadjusted model, higher zinc intake was significantly associated with lower stroke risk (OR = 0.925, 95% CI: 0.901 – 0.951, p < 0.00001). This association remained significant after adjusting for age, gender, and race (Model 1, OR = 0.946, 95% CI: 0.919 – 0.973, p = 0.00014) and additional covariates (Model 2, OR = 0.957, 95% CI: 0.930 – 0.985, *p* = 0.00302), suggesting a robust negative relationship between zinc intake and stroke risk, even after adjusting for potential confounders. The Generalized Additive Model (GAM) confirmed a negative linear relationship between zinc intake (0 - 20mg) and stroke risk, supporting findings from observational and GWAS studies. A logarithmic transformation of zinc intake further revealed a downward trend in stroke risk (**Supplementary Figure S1**). Stratified analyses by age, gender, race, income, education, smoking, and BMI showed a consistent significant negative association between zinc intake and stroke risk across all subgroups (OR < 1, *p* < 0.05). No significant interactions were observed among the subgroups, reinforcing the overall negative relationship between dietary zinc and stroke.3.3 Acquisition and analysis of zinc-autophagy-related targets in stroke.

**Table 1:** Characteristics of stroke and non-stroke groups.

Stroke	No (n=9293)	Yes (n=362)	P-value
Age	49.120 ± 17.416	64.787 ± 12.812	<0.001
TG	1.368 ± 1.197	1.425 ± 1.217	0.374
TC	4.946 ± 1.069	4.681 ± 1.206	<0.001
Sex			0.793
Male	4376 (47.089%)	173 (47.790%)	
Female	4917 (52.911%)	189 (52.210%)	
Race			<0.001
Hispanic	2336 (25.137%)	56 (15.470%)	
Non-Hispanic White	3940 (42.398%)	172 (47.514%)	
Non-Hispanic African American	1804 (19.412%)	108 (29.834%)	
Other Race - Including Multi-Racial	1213 (13.053%)	26 (7.182%)	
IPR			<0.001
Low-income	1948 (20.962%)	83 (22.928%)	
Middle-income	4953 (53.298%)	223 (61.602%)	
High-income	2392 (25.740%)	56 (15.470%)	
Edu			<0.001
Less than high school education	1991 (21.425%)	122 (33.702%)	
High school diploma	2050 (22.060%)	91 (25.138%)	
Higher than high school education	5252 (56.516%)	149 (41.160%)	
Smoke			<0.001
Non smokers	5280 (56.817%)	145 (40.055%)	
Former smokers	2254 (24.255%)	118 (32.597%)	
Current smokers	1759 (18.928%)	99 (27.348%)	
BMI			0.063
<18.5	138 (1.485%)	6 (1.657%)	
18.5 - 24	1972 (21.220%)	59 (16.298%)	
24 - 28	2469 (26.568%)	90 (24.862%)	
>28	4714 (50.726%)	207 (57.182%)	
Hypertension			<0.001
No	7729 (83.170%)	250 (69.061%)	
Yes	1564 (16.830%)	112 (30.939%)	
Diabetes			<0.001
No	7922 (85.247%)	267 (73.757%)	
Yes	1371 (14.753%)	95 (26.243%)	

**Figure 3:**
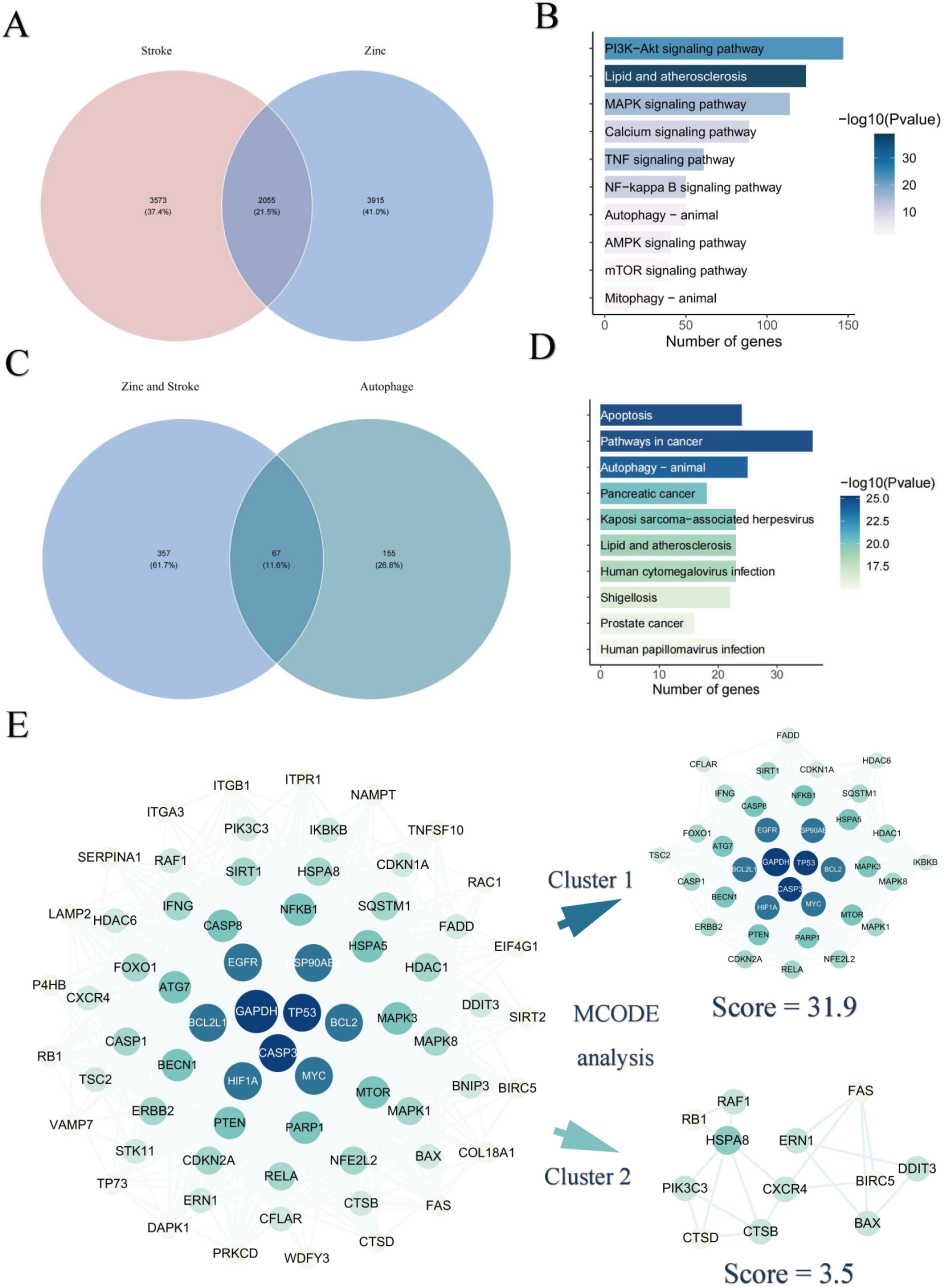
Identification of zinc–autophagy–stroke targets. **(A)** Overlap between zinc-related and stroke-related targets. **(B)** KEGG enrichment of overlapping targets. **(C)** Overlap among zinc-related, stroke-related, and autophagy-related targets. **(D)** KEGG enrichment of zinc–stroke–autophagy targets. **(E)** PPI network and MCODE analysis of overlapping targets between zinc, stroke and autophagy.

To investigate the molecular mechanisms connecting zinc and stroke, we performed To investigate the molecular connection between zinc and stroke, we performed pathway enrichment analysis and constructed a PPI network, identifying 2,055 overlapping targets associated with both zinc and stroke (**Figure 3A**). Enrichment analysis of these targets showed significant involvement in autophagy, MAPK signaling, lipid metabolism, atherosclerosis, and PI3K–Akt signaling (**Figure 3B**, **Supplementary Table S2**). We further focused on the role of autophagy in the mechanism of zinc-related stroke, identifying 67 zinc-autophagy-related targets in stroke (**Figure 3C**). Enrichment results of 67 targets revealed that autophagy and PI3K-Akt signaling were significantly enriched (**Figure 3D**, **Supplementary Table S4**). The PPI network of these 67 targets was highly interconnected, with GAPDH, TP53, CASP3, and RELA emerging as central genes (**Figure 3E**, **Supplementary Table S6**). MCODE analysis revealed two major target modules. Cluster 1 includes NFE2L2, BECN1, CASP8, and RELA, enriched in PI3K-Akt, MAPK, and NF-κB signaling pathways, implicating these genes’ roles in autophagy regulation, inflammation, and apoptosis. Cluster 2 contained CXCR4, ERN1 and DDIT3, linked to lysosomal autophagic degradation and DNA damage response pathways. GO enrichment results are provided in the **Supplementary Materials** (**Supplementary Figures S2, S3**, **Supplementary Tables S3**, **S5**).

### 3.4 Diagnostic model identified ZIARs as regulators of autophagy in stroke

**Figure 4:**
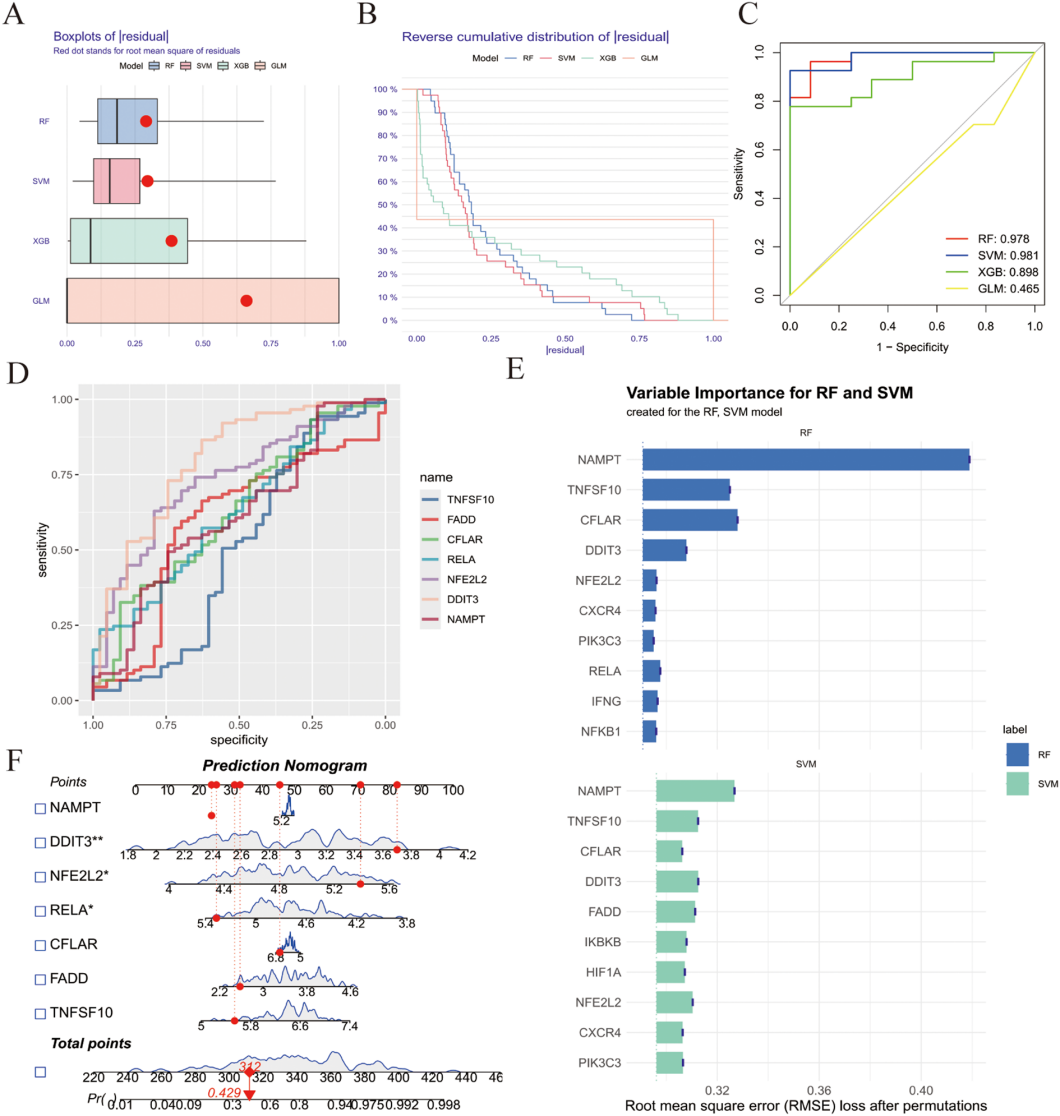
Machine learning–based diagnostic modeling for ZIARs. **(A)** Boxplots of residuals for four algorithms (RF, SVM, XGBoost, GLM). **(B)** Residual distribution plots. **(C)** ROC curves for all models. **(D)** Variable importance for the RF and SVM models. **(E)** ROC curves for the optimized SVM and RF models. **(F)** Feature importance and resulting nomogram for stroke risk. Asterisks = statistical significance: * P ≤ 0.05; ** P ≤ 0.01.

To further investigate the diagnostic value of ZIARs, we applied four machine learning algorithms (SVM, XGB, RF, and GLM) on the training set of 67 genes to investigate their diagnostic value. Residual boxplots showed significant differences in residual distributions among the four algorithms (**Figure 4A**). The SVM and RF models exhibited higher predictive accuracy than the others (**Figure 4B**), with AUC values of 0.981 and 0.978, respectively (**Figure 4C**). Considering their minimal residuals and high AUC, we chose SVM and RF as the optimal models for the diagnostic classifier. Both the SVM and RF models identified NAMPT, TNFSF10, RELA, NFE2L2, FADD, DDIT3, and CFLAR as influential regulators, namely ZIARs (**Figures 4D, E**). A diagnostic nomogram incorporating these genes was then constructed for stroke (**Figure 4F**).

### 3.5 ZIARs expression is strongly correlated with immune cell infiltration in stroke

**Figure 5:**
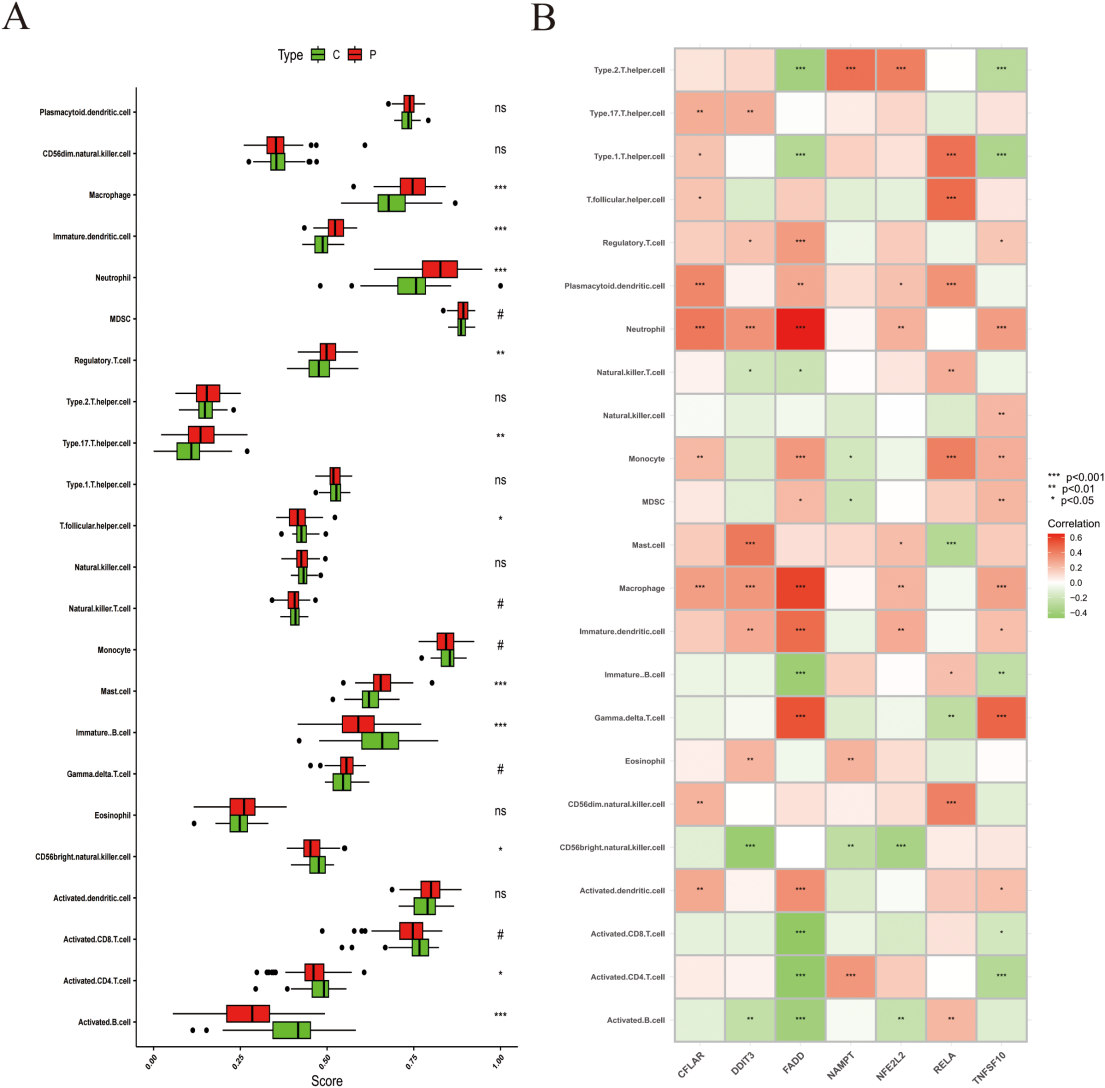
Immune cell infiltration analysis in stroke. **(A)** Infiltration scores for 22 immune cell types (boxplots: median and IQR). **(B)** Heatmap of Spearman correlations between ZIAR expression and immune cell infiltration scores. Asterisks = statistical significance: * P ≤ 0.05; ** P ≤ 0.01; *** P ≤ 0.001; ns = not significant (P > 0.05).

To investigate immune cell infiltration in stroke, we analyzed 22 immune cell types using the ssGSEA algorithm with leukocyte lineage gene sets curated by Charoentong et al (25) on PBMC transcriptomes. The results revealed higher enrichment scores for macrophages, dendritic cells, neutrophils, regulatory T cells, T helper 17 (Th17) cells, T follicular helper cells, and mast cells in stroke compared with controls (**Figure 5A**). Conversely, enrichment scores for immature B cells, natural killer cells, activated CD8 T cells, and activated B cells were lower. We further explored the relationship between ZIARs expression and peripheral immune cell signatures (**Figure 5B**). ZIAR expression showed strong correlations with immune cell patterns in stroke, indicating complex regulatory effects across leukocyte subgroups. Spearman correlation analysis across the 22 immune cell types and ZIARs identified that TNFSF10, RELA, NFE2L2, and CFLAR showed broadly positive correlations with multiple leukocyte lineage scores, whereas FADD, DDIT3, and NAMPT were negatively correlated. Notably, DDIT3, FADD, NFE2L2, and TNFSF10 correlated positively with neutrophils, macrophages, and immature dendritic cells. In contrast, FADD and TNFSF10 were negatively correlated with helper T cells (Th1/Th2), immature B cells, activated CD4 T cells, and activated B cells. These findings highlight the significant role of zinc in modulating the peripheral immune landscape in stroke, consistent with recognized systemic immune activation after ischemic stroke (33, 34).

### 3.6 Drug prediction targeting ZIARs in stroke

**Figure 6:**
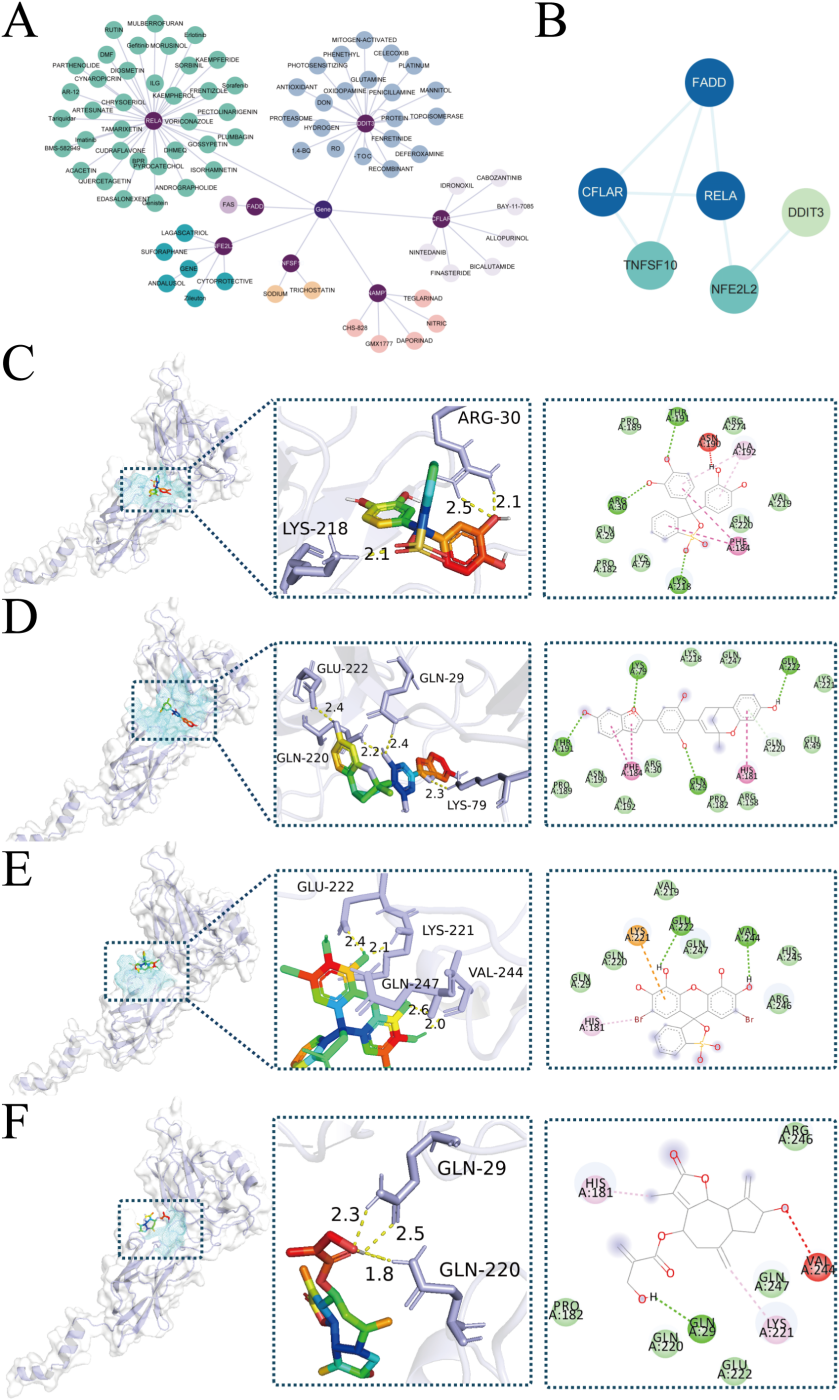
Drug-targets associations and molecular docking. **(A)** Network representation of predicted drugs and ZIARs. **(B)** Gene interaction network for ZIARs highlighting RELA’s centrality. **(C–F)** Docking poses of RELA with predicted compounds: **(C)** Pyrocatechol Violet, **(D)** Mulberrofurant H, **(E)** Bromopyrogallol Red, **(F)** Cynaropicrin.

We used the DGIdb database to identify potential therapeutic drugs for stroke focusing on ZIARs. A total of 82 drugs targeting these genes were identified (**Supplementary Table S7**). Our DGIdb query identified a total of 82 candidate drugs targeting the ZIAR genes (**Supplementary Table S7**). Notably, RELA had the most predicted drug interactions (36 compounds, e.g. Pyrocatechol Violet (PCV), Mulberrofurant H (MFH), Bromopyrogallol Red, Cynaropicrin, and DDIT3 had 23 (e.g. 6-Dimethylthioquinazolinone, DON). Other ZIARs had fewer associated compounds: CFLAR (7), NFE2L2 (6), NAMPT (5), TNFSF10 (3), and FADD (**Figure 6A**). The Gene interaction network for ZIARs showed that RELA is tightly interconnected with the other hub targets (**Figure 6B**). Given RELA’s central position, we performed molecular docking for top RELA-associated compounds: Pyrocatechol Violet, Mulberrofurant H, Bromopyrogallol Red, and Cynaropicrin. Docking results indicated that Pyrocatechol Violet (binding energy = –7.9 kcal/mol) forms stable contacts with RELA (e.g., ARG30 and LYS218) via van der Waals forces and hydrogen bonds (**Figure 6C**). Similarly, Mulberrofurant H (–8.1 kcal/mol) interacts with residues LYS79, GLN29, and GLUee222 through notable π–π stacking and H-bonds, indicating strong affinity for RELA (**Figure 6D**). Bromopyrogallol Red (Binding Energy = -7.6 kcal/mol) exhibits multiple favorable interactions, including Pi-Cation and hydrogen bonds, with residues such as GLU222 and LYS221 (**Figure 6E**). Cynaropicrin (Binding Energy = -6.3 kcal/mol) forms key hydrogen bonds with GLN29 and GLN220, and unfavorable acceptor-acceptor interactions with VAL244 (**Figure 6F**).

### 3.7 Zinc alleviates cell injury in stroke model via autophagy modulation

**Figure 7:**
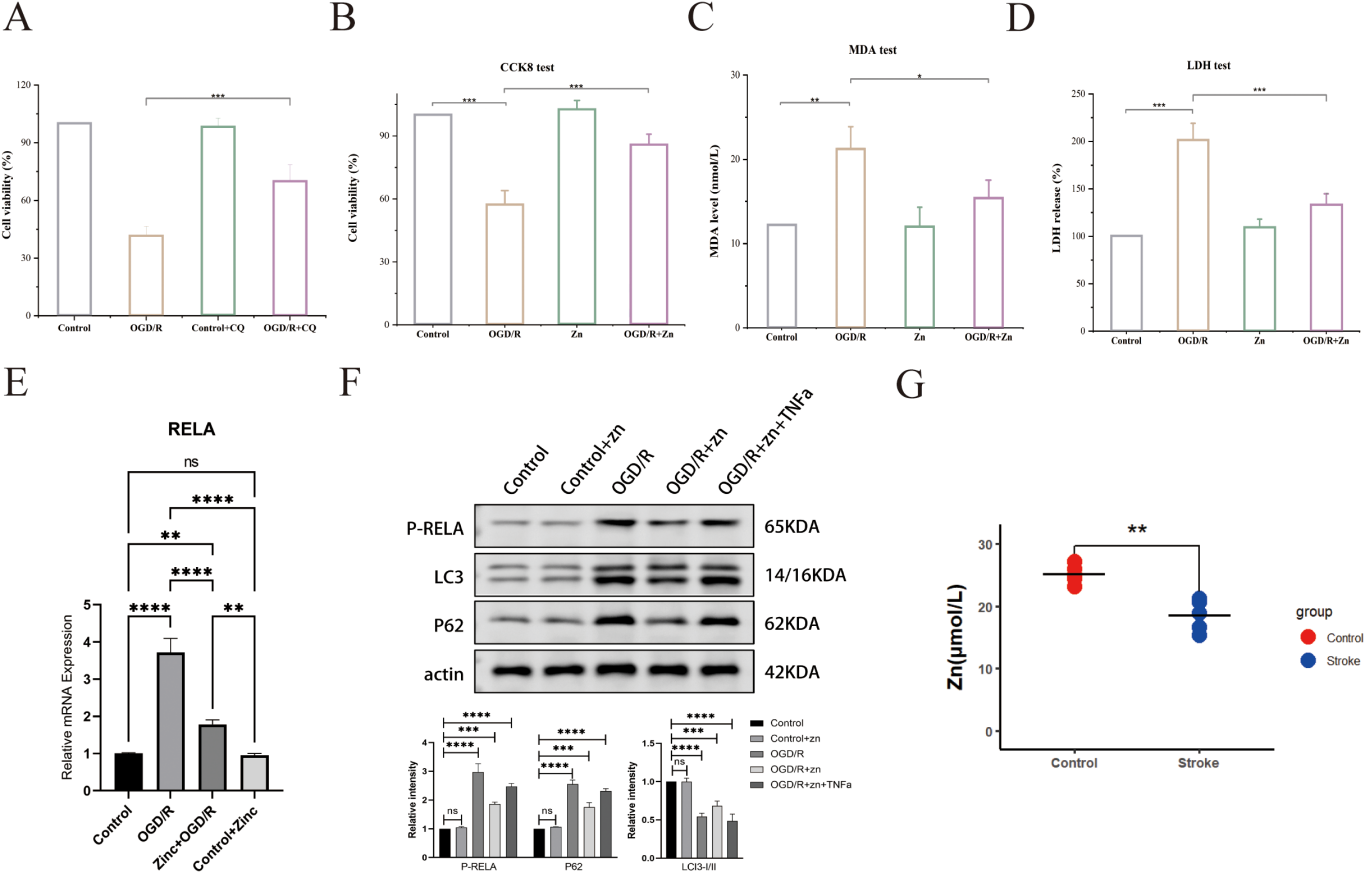
Zinc’s effects on cell viability and biomarkers in the stroke model. **(A)** Cell viability (CCK - 8) in CQ treatment groups. **(B)** CCK - 8 assay for cell survival. **(C)** MDA assay for oxidative stress. **(D)** LDH activity assay. **(E)** qPCR analysis of RELA mRNA. **(F)** Western blot for LC3 and p62. **(G)** Serum zinc levels in healthy controls and patients with stroke. Asterisks indicate statistical significance: * P ≤ 0.05; ** P ≤ 0.01; *** P ≤ 0.001; **** P ≤ 0.0001; ns = not significant (P > 0.05).

To investigate the role of autophagy in zinc’s neuroprotective effects, we used chloroquine (CQ), an autophagy inhibitor. CQ treatment significantly reduced zinc’s protective effect against oxidative stress-induced cell injury, leading to decreased cell survival (**Figure 7A**). This highlights the critical role of autophagy in zinc-mediated neuroprotection. Cell viability assays (CCK - 8) demonstrated that the OGD/R group had significantly reduced cell survival compared to the control and zinc-treated groups. Zinc supplementation improved cell proliferation compared to the OGD/R group, though survival remained lower than control levels (**Figure 7B**). Oxidative stress, assessed by MDA levels, was significantly elevated in the OGD/R group. Zinc treatment partially alleviated this increase, though MDA levels did not fully return to baseline control levels (**Figure 7C**). Lactate dehydrogenase (LDH) activity assays confirmed severe cell damage following OGD/R treatment. Zinc supplementation reduced cell damage, but protection was not complete, as LDH levels remained higher than control values (**Figure 7D**). qPCR results revealed that OGD/R markedly upregulated RELA mRNA expression, an effect significantly attenuated by zinc pretreatment to near-control levels, while zinc alone had no impact under normoxia (**Figure 7E**). Consistently, Western blot analysis showed that zinc reduced OGD/R-induced RELA phosphorylation, an effect reversed by TNFα, indicating NF-κB pathway suppression. Decreased P62 and LC3-II/I ratio further suggested reduced autophagosome accumulation and enhanced autophagic degradation (**Figure 7F**). Serum zinc levels were significantly lower in stroke patients than in healthy controls, consistent with previous reports (**Figure 7G**).

## 4 Discussion

Stroke is largely driven by neuroinflammation and dysregulated autophagy (35). Zinc is an essential micronutrient with antioxidant and immunomodulatory properties (36). However, its causal role in stroke protection and the underlying mechanisms were not well established. Our analyses indicate that higher zinc status may reduce stroke risk by modulating autophagy and post-stroke immune responses. Specifically, in the GWAS analysis, genetically higher zinc levels were associated with significantly lower stroke risk (OR = 0.141, P = 0.013). In the NHANES cohort, greater dietary zinc intake correlated with lower stroke prevalence in a dose-dependent manner (OR = 0.957, P = 0.00302). Integrated bioinformatics and diagnostic modeling further identified key zinc-related genes enriched in the autophagy pathway, and these findings were validated by cellular experiments. We found that ZIARs influence the pattern of immune cell infiltration in stroke. Furthermore, we predicted candidate drugs targeting ZIARs and performed molecular docking to validate their binding. Notably, the neuroprotective effect of zinc involved ZIAR-mediated autophagy pathways and a reduction of excessive immune cell infiltration.

To elucidate underlying mechanisms, we performed KEGG and GO enrichment analyses and found that zinc-associated genes in stroke are enriched in autophagy, NF-κB, and apoptosis pathways. Autophagy is a key process in ischemic stroke (2). In moderation, autophagy protects neurons by clearing damaged organelles and proteins, but excessive autophagy can cause cell death. Indeed, neuronal survival depends on maintaining autophagic flux within an optimal range: insufficient flux allows toxic aggregates to accumulate, whereas excessive flux triggers autophagy-dependent cell death (37, 38). This concept explains autophagy’s dual roles in ischemic injury and supports the idea that zinc’s neuroprotection comes from preventing excessive autophagy-mediated neuronal loss. These findings support the hypothesis that zinc prevents excessive autophagy−mediated neuronal loss. To experimentally validate the role of zinc and autophagy in stroke, we employed an OGD/R cellular model to simulate ischemic conditions. Consistent with expectations, OGD/R caused substantial neuronal injury, evidenced by elevated MDA and LDH levels, whereas zinc pretreatment markedly attenuated both oxidative damage and cytotoxicity (CCK - 8 viability). At the molecular level, OGD/R led to autophagosome accumulation – indicated by increased LC3-II and p62 – signifying impaired autophagic flux (39). Notably, zinc pre-treatment normalized these autophagy markers, suggesting that zinc helps maintain autophagic homeostasis. Furthermore, adding CQ (an autophagy inhibitor) abolished zinc’s neuroprotective effects: cell viability dropped and injury markers rose back to OGD/R-alone levels. This confirms that autophagy is a key mediator of zinc’s protective effect in our model.

Notably, the effects of zinc after ischemia are highly context-dependent, varying with dose, timing, and subcellular localization. Our human data (GWAS, NHANES, and PBMC transcriptomics) reflect systemic zinc status, whereas studies reporting zinc-induced neurotoxicity typically involve localized neuronal or synaptic zinc accumulation in the ischemic core, which aggravates oxidative stress and inflammatory responses (6, 7). In our OGD/R cell model, a moderate zinc level restored autophagic flux and suppressed RELA/NF-κB activation. This aligns with prior evidence that controlled zinc can enhance lysosomal-autophagy function (40), and it mirrors our readouts in Zn-treated cells (lower p-RELA, normalized LC3-II/I and p62; **Figures 7E, F**). In summary, physiological levels of zinc appear neuroprotective, whereas compartmentalized zinc overload is detrimental, which reconciles the seemingly contradictory effects observed in different contexts.

To assess the diagnostic potential of core molecular signatures in stroke, we established a machine learning–based model that identified seven key genes, termed ZIARs. Compared with previous studies (41), our model achieved an AUC of 0.98 in the training set, indicating improved accuracy and thus offers a powerful tool for stroke patient classification. The key genes encompass critical pathways such as autophagy (e.g. NFE2L2, DDIT3, NAMPT) (42, 43), apoptosis (e.g. CFLAR, FADD) and NF-κB signaling (e.g. RELA, TNFSF10) (44, 45), suggesting a multifactorial involvement of these pathways in stroke pathophysiology.

To explore the expression changes of immune cells in stroke pathophysiology, we used ssGSEA to perform immune infiltration analysis. Zinc status is known to act as a “gatekeeper” of immune function, with zinc deficiency increasing the production of pro-inflammatory cytokines and impairing immune cell function (46). Our results showed that stroke was associated with heightened infiltration of innate immune cells (e.g. neutrophils and macrophages) and dendritic cells, along with a reduction in certain adaptive lymphocytes (e.g. regulatory T cells, activated T cells, and B cells) compared to controls. This shift toward a pro-inflammatory innate immune milieu is consistent with the acute immunological response after stroke. Macrophages and neutrophils infiltrate the injured brain parenchyma earlier and more abundantly than lymphocytes, exacerbating tissue damage (47). Immune infiltration analysis of the key genes revealed that ZIARs may mediate the pro-inflammatory innate immune environment in stroke, suggesting a role for zinc in modulating post-stroke inflammation. Taken together, we infer zinc may help shape a more favorable immune microenvironment in stroke and limit secondary brain injury.

RELA emerged as a central hub in our zinc–stroke network and is notably the central subunit of the NF-κB transcription factor complex (48). It is highly expressed in key infiltrating immune cells (monocytes and CD56^dim NK cells in particular) during stroke. Functionally, RELA orchestrates inflammatory gene expression in the innate immune system and also acts upstream to initiate autophagy (49). Its critical importance is highlighted by the fact that RELA deletion in mice causes embryonic lethality due to massive liver apoptosis and impaired lymphocyte activation, underscoring RELA’s essential role in immune homeostasis (50). qPCR analysis revealed that zinc suppresses OGD/R-induced RELA mRNA upregulation, and Western blot confirmed reduced RELA phosphorylation with restored autophagic flux, as indicated by decreased p62 and normalized LC3-II/I ratio, consistent with prior reports (2, 40, 51). Together, these findings highlight RELA as a downstream effector linking zinc-mediated autophagy modulation to NF-κB–driven inflammation. We therefore propose that RELA hyperactivation during stroke disrupts autophagic flux, sustaining proinflammatory cytokine release and driving infiltration and activation of monocytes and CD56^dim NK cells. NF-κB signaling drives monocyte chemokine production (52) and exerts cytotoxic effects in CD56^dim NK cells (53), facilitating their invasion into injured brain tissue. This RELA hyperactivation likely enhances monocyte chemotaxis across a compromised blood–brain barrier and intensifies CD56^dim NK cell cytotoxicity, exacerbating neuronal death and tissue damage, as shown by previous studies (54, 55). These findings align with our observations of an inflammatory shift in the innate infiltrate. Because the immune scores were derived from PBMC transcriptomes using a lineage-level leukocyte signature (25), our inferences pertain to the peripheral immune landscape accompanying stroke rather than brain-resident glia. This distinction is consistent with human studies documenting robust systemic immune remodeling after stroke (33, 34). Future work integrating CNS single-cell–derived neuroinflammation signatures will refine tissue-specific inferences. Collectively, these findings suggest that restoring zinc homeostasis or enhancing autophagy may interrupt the deleterious RELA−driven inflammatory cascade and improve outcomes in stroke patients. Consistent with this notion, pharmacological inhibition of NF-κB signaling has been shown to reduce infarct volume and improve neurological outcomes in rodent models of ischemic stroke (56–58). These *in vivo* data reinforce the therapeutic relevance of targeting RELA/NF-κB, positioning zinc not only as a physiological modulator but also as a potential adjunct to NF-κB–directed interventions.

In summary, our integrative, multi-level analysis highlights zinc as a critical modulator of stroke pathogenesis. Both genetic and dietary zinc sufficiency were associated with reduced stroke risk and milder injury, underscoring the potential of zinc-targeted interventions for stroke prevention and therapy. Mechanistically, zinc’s neuroprotective effects stem from its coordination of autophagy and modulation of immune responses via ZIARs – particularly through RELA-mediated pathways. Overall, this work reveals the importance of the zinc–autophagy–immune axis in stroke and lays a foundation for developing ZIAR-targeted therapeutic strategies.

## 5 Conclusion

In conclusion, this study reveals that elevated zinc levels can reduce the risk of stroke by modulating the zinc-autophagy-immune axis. Specifically, we identified ZIARs as potential mediators of stroke pathophysiology, acting through the regulation of immune cell infiltration and autophagy. Collectively, our findings establish a new mechanistic framework for understanding stroke, and suggest that zinc-based or ZIAR-targeted interventions could be promising strategies for stroke prevention and neuroprotection. It is valuable to carry out pre - clinical zinc supplementation trials and gene knockout experiments for ZIARs in future studies.

## Data Availability

The original contributions presented in the study are included in the article/**Supplementary Material**. Further inquiries can be directed to the corresponding authors.
