# Supplementary material for: Zinc alleviates stroke development through autophagy-mediated modulation of immune microenvironment
Source: Front Immunol. 2025 Sep 3;16:1666225. doi: 10.3389/fimmu.2025.1666225 (PMC12440758; doi:10.3389/fimmu.2025.1666225)
Supplement: Supplementary file 1 [file DataSheet1.docx]

## Supplementary Figures and Tables

## 1.1 Supplementary Figures
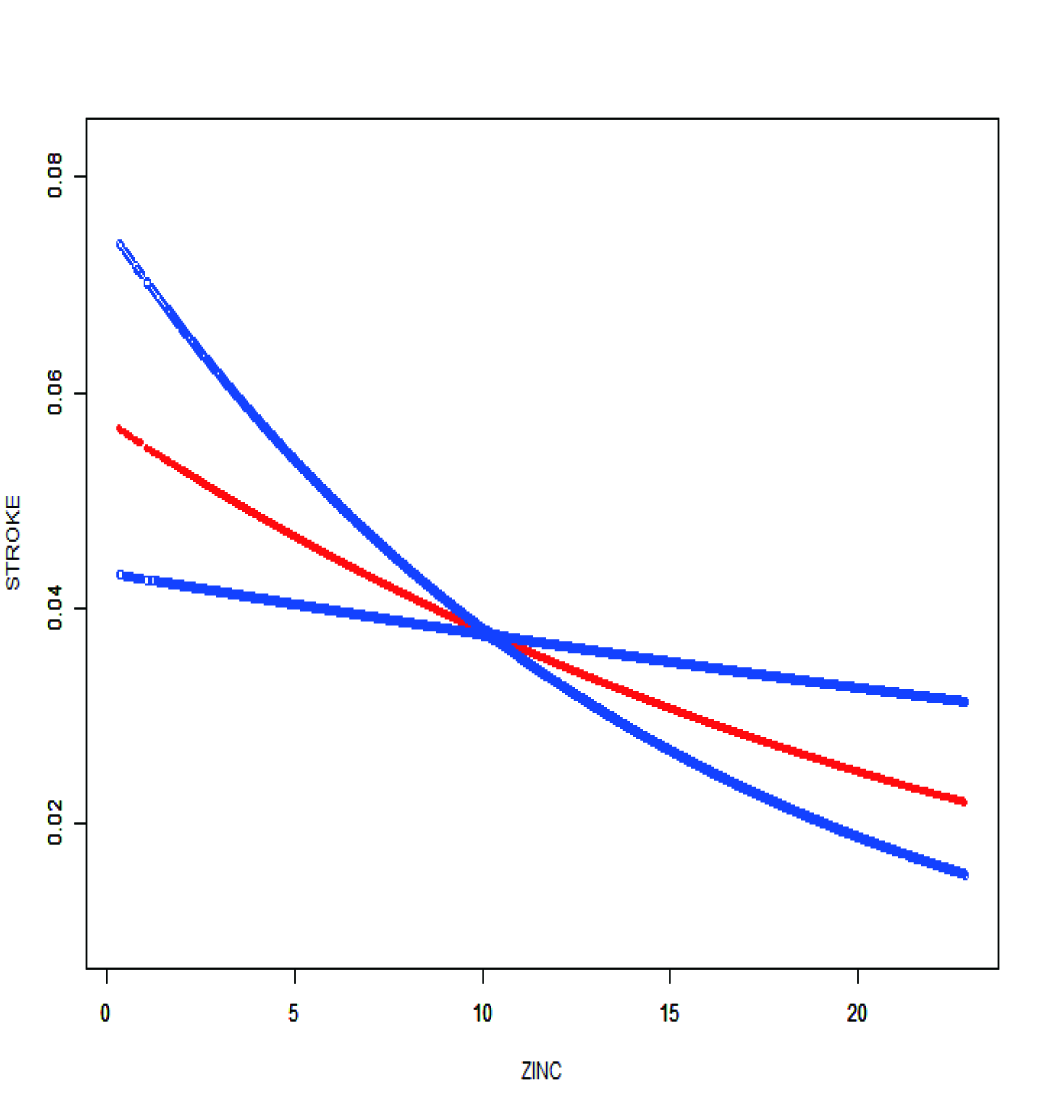


Figure S1 Smooth curve of dietary zinc intake and stroke.


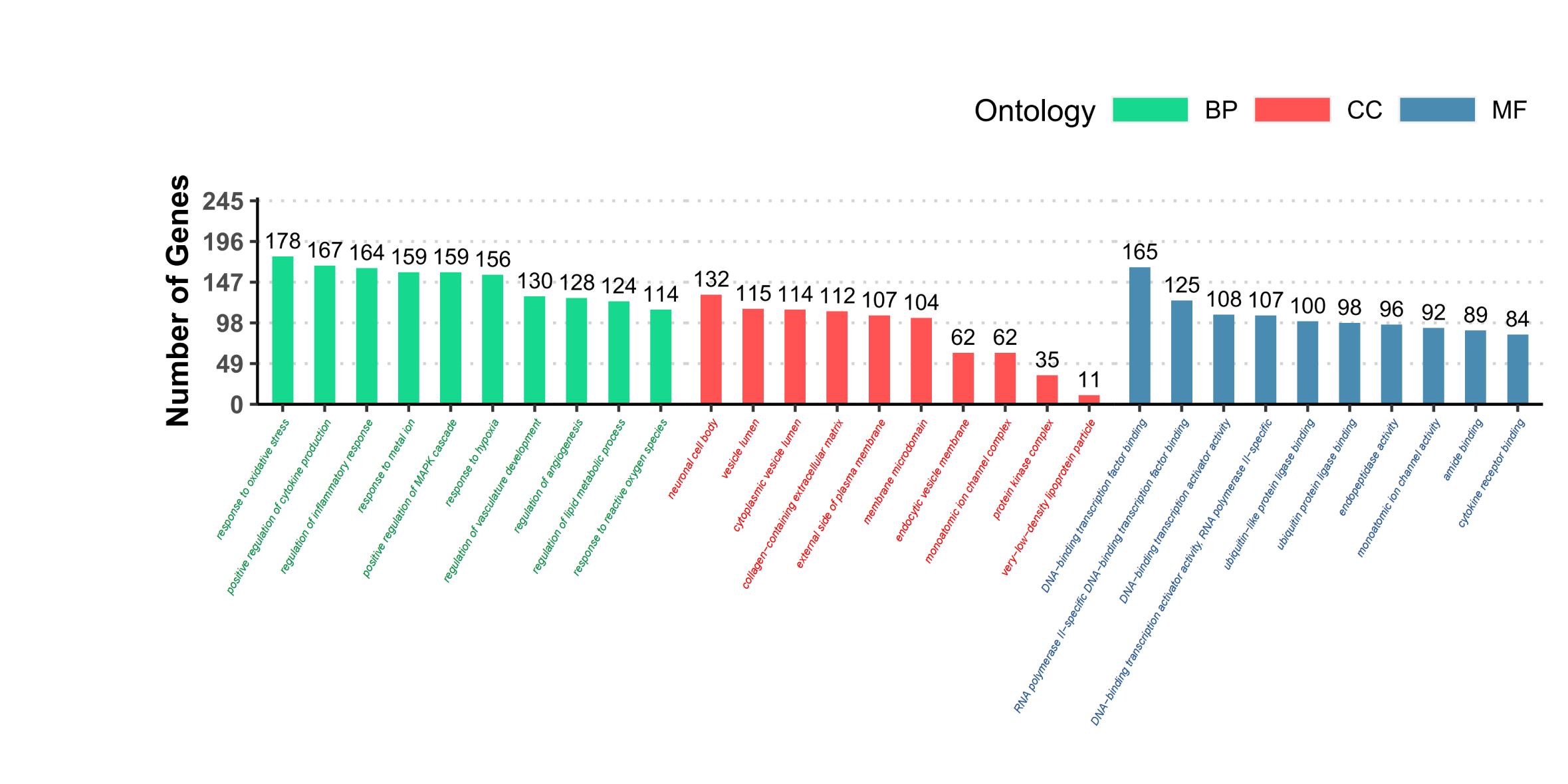


Figure S2 GO analysis of the intersecting genes between zinc and stroke.


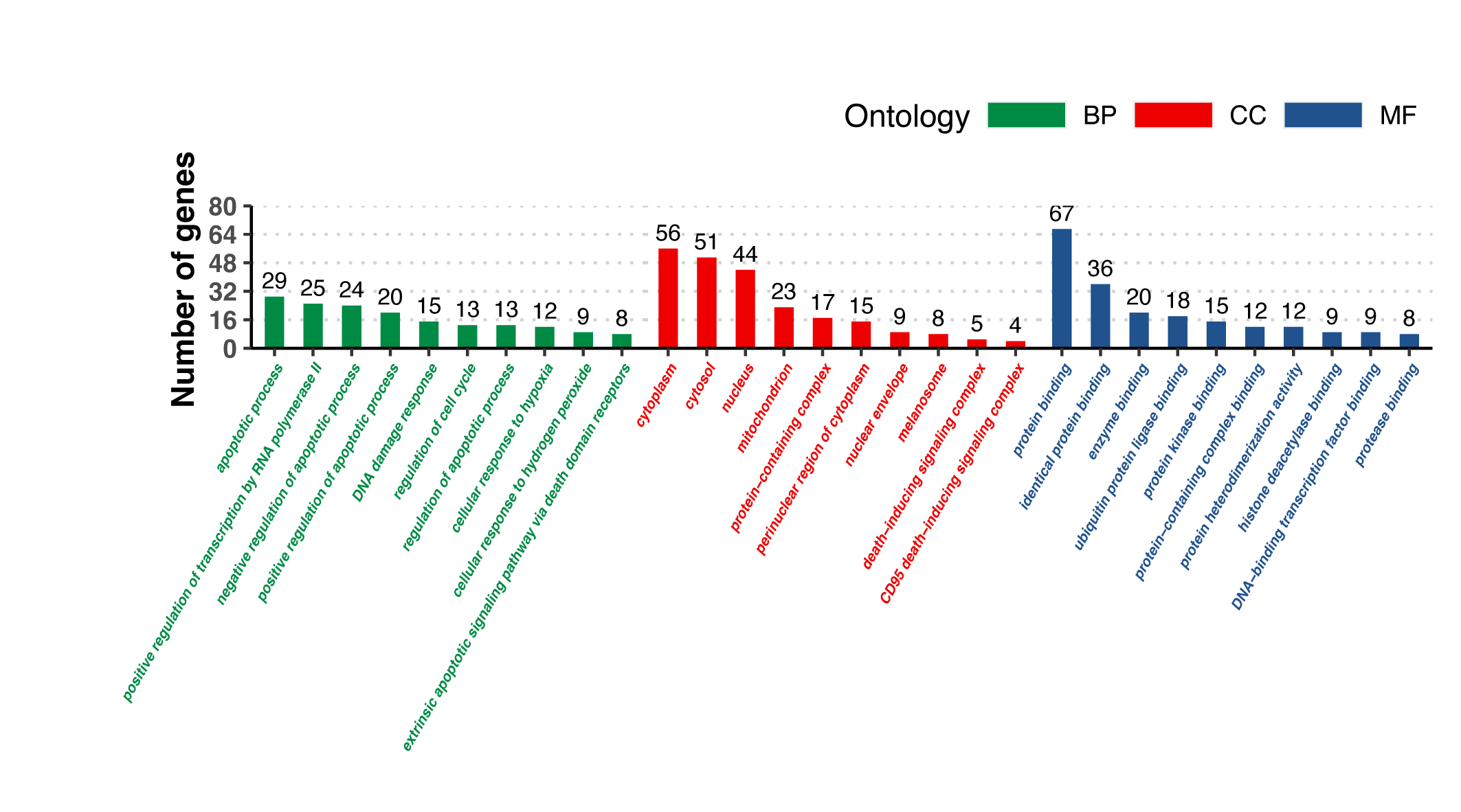


Figure S3 GO analysis of the key genes

1.2 Supplementary Table
Table S1 Summary of total elements GWAS analysis results.

| Zinc | CES.TRANS | id.exposure | id.outcome | outcome | exposure | method | nsnp | b | se | pval | lo_ci | up_ci | or |
| --- | --- | --- | --- | --- | --- | --- | --- | --- | --- | --- | --- | --- | --- |
| 1 |  | OgnxmU | PCRKSr | outcome | exposure | MR Egger | 9 | -3.823837602 | 1.358015178 | 0.025930787 | -6.485547351 | -1.162127854 | 0.021843812 |
| 2 |  | OgnxmU | PCRKSr | outcome | exposure | Weighted median | 9 | -1.991099196 | 0.263689021 | 4.32E-14 | -2.507929677 | -1.474268715 | 0.136545253 |
| 3 |  | OgnxmU | PCRKSr | outcome | exposure | Inverse variance weighted | 9 | -2.977188837 | 0.549475599 | 6.02E-08 | -4.054161012 | -1.900216662 | 0.050935822 |
| 4 |  | OgnxmU | PCRKSr | outcome | exposure | Simple mode | 9 | -2.848630894 | 0.47688422 | 0.00033313 | -3.783323964 | -1.913937824 | 0.05792357 |
| 5 |  | OgnxmU | PCRKSr | outcome | exposure | Weighted mode | 9 | -2.275603243 | 0.312010722 | 8.44E-05 | -2.887144257 | -1.664062229 | 0.102734916 |
| Zinc | CES.EUR | id.exposure | id.outcome | outcome | exposure | method | nsnp | b | se | pval | lo_ci | up_ci | or |
| 1 |  | OgnxmU | C2jOyF | outcome | exposure | MR Egger | 9 | -1.90612502 | 2.027861568 | 0.378528982 | -5.880733692 | 2.068483652 | 0.148655308 |
| 2 |  | OgnxmU | C2jOyF | outcome | exposure | Weighted median | 9 | 0.102160816 | 0.138345546 | 0.46024303 | -0.168996455 | 0.373318086 | 1.107561571 |
| 3 |  | OgnxmU | C2jOyF | outcome | exposure | Inverse variance weighted | 9 | -1.961686231 | 0.79053452 | 0.01308424 | -3.51113389 | -0.412238571 | 0.140621101 |
| 4 |  | OgnxmU | C2jOyF | outcome | exposure | Simple mode | 9 | -0.867315047 | 0.422577489 | 0.074226385 | -1.695566926 | -0.039063167 | 0.420077926 |
| 5 |  | OgnxmU | C2jOyF | outcome | exposure | Weighted mode | 9 | 0.049108057 | 0.109062264 | 0.664463727 | -0.16465398 | 0.262870094 | 1.05033384 |
| Iron | AIS.TRANS | id.exposure | id.outcome | outcome | exposure | method | nsnp | b | se | pval | lo_ci | up_ci | or |
| 1 |  | myP2jf | ALiqn3 | outcome | exposure | MR Egger | 4 | 0.002202707 | 0.021944743 | 0.929202136 | -0.040808988 | 0.045214403 | 1.002205135 |
| 2 |  | myP2jf | ALiqn3 | outcome | exposure | Weighted median | 4 | 0.043399975 | 0.000494135 | 0 | 0.042431469 | 0.04436848 | 1.044355527 |
| 3 |  | myP2jf | ALiqn3 | outcome | exposure | Inverse variance weighted | 4 | 0.044815353 | 0.014219707 | 0.001623581 | 0.016944727 | 0.072685979 | 1.045834731 |
| 4 |  | myP2jf | ALiqn3 | outcome | exposure | Simple mode | 4 | 0.073797282 | 0.001393683 | 1.48E-05 | 0.071065662 | 0.076528901 | 1.076588539 |
| 5 |  | myP2jf | ALiqn3 | outcome | exposure | Weighted mode | 4 | 0.030832105 | 0.000638108 | 1.95E-05 | 0.029581414 | 0.032082796 | 1.031312337 |
| Vitamin A | SVS.EUR | id.exposure | id.outcome | outcome | exposure | method | nsnp | b | se | pval | lo_ci | up_ci | or |
| 1 |  | ew35Xa | m3PlD5 | outcome | exposure | MR Egger | 12 | -9.411566878 | 13.30524835 | 0.495498341 | -35.48985365 | 16.66671989 | 8.18E-05 |
| 2 |  | ew35Xa | m3PlD5 | outcome | exposure | Weighted median | 12 | 1.91749941 | 0.560169616 | 0.000619183 | 0.819566963 | 3.015431856 | 6.803923354 |
| 3 |  | ew35Xa | m3PlD5 | outcome | exposure | Inverse variance weighted | 12 | 11.02400833 | 4.506796641 | 0.014441689 | 2.190686915 | 19.85732975 | 61329.01464 |
| 4 |  | ew35Xa | m3PlD5 | outcome | exposure | Simple mode | 12 | 5.312083827 | 1.285607587 | 0.001666566 | 2.792292956 | 7.831874698 | 202.772331 |
| 5 |  | ew35Xa | m3PlD5 | outcome | exposure | Weighted mode | 12 | 1.943786505 | 0.452202433 | 0.001259143 | 1.057469738 | 2.830103273 | 6.985150267 |
| Sodium | LAS.EUR | id.exposure | id.outcome | outcome | exposure | method | nsnp | b | se | pval | CI_lower | CI_upper | OR |
| 1 |  | qUPpMV | SOvyI6 | outcome | exposure | MR Egger | 65 | 0.668786753 | 0.984615473 | 0.499475996 | 0.283353633 | 13.44534674 | 1.951867785 |
| 2 |  | qUPpMV | SOvyI6 | outcome | exposure | Weighted median | 65 | 0.917352971 | 0.358358646 | 0.010471013 | 1.239824732 | 5.051756058 | 2.502657009 |
| 3 |  | qUPpMV | SOvyI6 | outcome | exposure | Inverse variance weighted | 65 | 0.500376697 | 0.24735253 | 0.043080431 | 1.015687515 | 2.67831444 | 1.649342456 |
| 4 |  | qUPpMV | SOvyI6 | outcome | exposure | Simple mode | 65 | 0.745618803 | 0.89474552 | 0.407757603 | 0.36491807 | 12.17421295 | 2.107745311 |
| 5 |  | qUPpMV | SOvyI6 | outcome | exposure | Weighted mode | 65 | 1.236007385 | 0.754617923 | 0.106345194 | 0.784237208 | 15.10549392 | 3.441844038 |
| Cobalt | SVS.TRANS | id.exposure | id.outcome | outcome | exposure | method | nsnp | b | se | pval | CI_lower | CI_upper | OR |
| 1 |  | Bhiots | KLYftq | outcome | exposure | MR Egger | 3 | -0.348659594 | 0.245649577 | 0.390741894 | 0.435991399 | 1.142037076 | 0.705633291 |
| 2 |  | Bhiots | KLYftq | outcome | exposure | Weighted median | 3 | -0.08920898 | 0.057336495 | 0.11973549 | 0.817431224 | 1.023441072 | 0.914654409 |
| 3 |  | Bhiots | KLYftq | outcome | exposure | Inverse variance weighted | 3 | -0.096044843 | 0.04505839 | 0.033042703 | 0.831636955 | 0.992299404 | 0.908423279 |
| 4 |  | Bhiots | KLYftq | outcome | exposure | Simple mode | 3 | -0.06984663 | 0.080005417 | 0.474707956 | 0.797193976 | 1.090857395 | 0.932536832 |
| 5 |  | Bhiots | KLYftq | outcome | exposure | Weighted mode | 3 | -0.127687114 | 0.079147169 | 0.248021261 | 0.753658776 | 1.027821318 | 0.880128716 |
| Cobalt | SVS.EUR | id.exposure | id.outcome | outcome | exposure | method | nsnp | b | se | pval | CI_lower | CI_upper | OR |
| 1 |  | Bhiots | KLYftq | outcome | exposure | MR Egger | 3 | -0.348659594 | 0.245649577 | 0.390741894 | 0.435991399 | 1.142037076 | 0.705633291 |
| 2 |  | Bhiots | KLYftq | outcome | exposure | Weighted median | 3 | -0.08920898 | 0.057336495 | 0.11973549 | 0.817431224 | 1.023441072 | 0.914654409 |
| 3 |  | Bhiots | KLYftq | outcome | exposure | Inverse variance weighted | 3 | -0.096044843 | 0.04505839 | 0.033042703 | 0.831636955 | 0.992299404 | 0.908423279 |
| 4 |  | Bhiots | KLYftq | outcome | exposure | Simple mode | 3 | -0.06984663 | 0.080005417 | 0.474707956 | 0.797193976 | 1.090857395 | 0.932536832 |
| 5 |  | Bhiots | KLYftq | outcome | exposure | Weighted mode | 3 | -0.127687114 | 0.079147169 | 0.248021261 | 0.753658776 | 1.027821318 | 0.880128716 |
| Cobalt | AIS.TRANS | id.exposure | id.outcome | outcome | exposure | method | nsnp | b | se | pval | CI_lower | CI_upper | OR |
| 1 |  | Bhiots | Evc0QU | outcome | exposure | MR Egger | 3 | 0.009286823 | 0.105174911 | 0.943932619 | 0.821308892 | 1.240394716 | 1.00933008 |
| 2 |  | Bhiots | Evc0QU | outcome | exposure | Weighted median | 3 | -0.047568712 | 0.021820474 | 0.029257196 | 0.913623387 | 0.995210922 | 0.953544951 |
| 3 |  | Bhiots | Evc0QU | outcome | exposure | Inverse variance weighted | 3 | -0.055092304 | 0.016161509 | 0.000652352 | 0.916889 | 0.976856274 | 0.946397787 |
| 4 |  | Bhiots | Evc0QU | outcome | exposure | Simple mode | 3 | -0.043372838 | 0.025556586 | 0.231765124 | 0.910771036 | 1.006740688 | 0.957554311 |
| 5 |  | Bhiots | Evc0QU | outcome | exposure | Weighted mode | 3 | -0.044343705 | 0.022160452 | 0.183363478 | 0.915964024 | 0.999091195 | 0.956625105 |
| Cobalt | AIS.EUR | id.exposure | id.outcome | outcome | exposure | method | nsnp | b | se | pval | CI_lower | CI_upper | OR |
| 1 |  | Bhiots | 6qTk1v | outcome | exposure | MR Egger | 3 | -0.078065108 | 0.125195346 | 0.645050513 | 0.723649597 | 1.182129851 | 0.924904206 |
| 2 |  | Bhiots | 6qTk1v | outcome | exposure | Weighted median | 3 | -0.06724846 | 0.023670702 | 0.004497234 | 0.892576517 | 0.979362055 | 0.934962872 |
| 3 |  | Bhiots | 6qTk1v | outcome | exposure | Inverse variance weighted | 3 | -0.064457362 | 0.018721485 | 0.00057538 | 0.903796122 | 0.972618606 | 0.93757609 |
| 4 |  | Bhiots | 6qTk1v | outcome | exposure | Simple mode | 3 | -0.08162805 | 0.032246537 | 0.127001031 | 0.865168267 | 0.981743863 | 0.92161469 |
| 5 |  | Bhiots | 6qTk1v | outcome | exposure | Weighted mode | 3 | -0.078714973 | 0.031123299 | 0.127185038 | 0.869604613 | 0.982442651 | 0.924303338 |
| Cobalt | AS.TRANS | id.exposure | id.outcome | outcome | exposure | method | nsnp | b | se | pval | CI_lower | CI_upper | OR |
| 1 |  | Bhiots | fJNRi6 | outcome | exposure | MR Egger | 3 | 0.017772869 | 0.085634722 | 0.869723655 | 0.860646707 | 1.203960963 | 1.017931746 |
| 2 |  | Bhiots | fJNRi6 | outcome | exposure | Weighted median | 3 | -0.042784314 | 0.020247043 | 0.034590993 | 0.920840427 | 0.99690469 | 0.95811802 |
| 3 |  | Bhiots | fJNRi6 | outcome | exposure | Inverse variance weighted | 3 | -0.050917244 | 0.015218485 | 0.000820604 | 0.922428443 | 0.979131804 | 0.950357315 |
|  |  |  |  |  |  |  |  |  |  |  |  |  |  |
| 4 |  | Bhiots | fJNRi6 | outcome | exposure | Simple mode | 3 | -0.040793169 | 0.023005181 | 0.218194418 | 0.917701299 | 1.004306231 | 0.960027673 |
| 5 |  | Bhiots | fJNRi6 | outcome | exposure | Weighted mode | 3 | -0.041326474 | 0.022124247 | 0.202726578 | 0.91879707 | 1.002039125 | 0.959515822 |
| Cobalt | AIS.EUR | id.exposure | id.outcome | outcome | exposure | method | nsnp | b | se | pval | CI_lower | CI_upper | OR |
| 1 |  | Bhiots | fJNRi6 | outcome | exposure | MR Egger | 3 | 0.017772869 | 0.085634722 | 0.869723655 | 0.860646707 | 1.203960963 | 1.017931746 |
| 2 |  | Bhiots | fJNRi6 | outcome | exposure | Weighted median | 3 | -0.042784314 | 0.020247043 | 0.034590993 | 0.920840427 | 0.99690469 | 0.95811802 |
| 3 |  | Bhiots | fJNRi6 | outcome | exposure | Inverse variance weighted | 3 | -0.050917244 | 0.015218485 | 0.000820604 | 0.922428443 | 0.979131804 | 0.950357315 |
| 4 |  | Bhiots | fJNRi6 | outcome | exposure | Simple mode | 3 | -0.040793169 | 0.023005181 | 0.218194418 | 0.917701299 | 1.004306231 | 0.960027673 |
| 5 |  | Bhiots | fJNRi6 | outcome | exposure | Weighted mode | 3 | -0.041326474 | 0.022124247 | 0.202726578 | 0.91879707 | 1.002039125 | 0.959515822 |
| Zinc reverse GWAS analysis results | | | | | | | | | | | | | |
| Sekenium | LAS.TRANS | id.exposure | id.outcome | outcome | exposure | method | nsnp | b | se | pval | CI_lower | CI_upper | OR |
| 1 |  | f0pfBc | k3pu1a | outcome | exposure | Inverse variance weighted | 2 | -0.228461826 | 0.10817392 | 0.034687633 | 0.643725614 | 0.983693471 | 0.795756674 |
| Sekenium | LAS.EUR | id.exposure | id.outcome | outcome | exposure | method | nsnp | b | se | pval | CI_lower | CI_upper | OR |
| 1 |  | f0pfBc | 9lPshy | outcome | exposure | Inverse variance weighted | 2 | -0.357144797 | 0.153729058 | 0.020167905 | 0.517651133 | 0.945694366 | 0.69967118 |

Table S2 KEGG pathway of zinc-stroke-related genes.

| ID | Description | Count | PValue | geneID |
| --- | --- | --- | --- | --- |
| hsa04151 | PI3K-Akt signaling pathway | 147 | 3.79E-25 | TP53/MDM2/IKBKG/INS/IL6/CREB1/BRCA1/VTN/IGF1R/CDKN1A/IL2RA/BDNF/GH1/IGF1/EGFR/MYC/MAPK1/IGF2/NR4A1/IFNA2/IL2/EPO/IKBKB/NGFR/TLR4/NTRK2/BCL2/CCND1/GDNF/FN1/PRL/NOS3/VEGFA/NTRK1/NFKB1/HRAS/GHR/AKT1/EGF/CHUK/PIK3CA/PTEN/FGFR1/INSR/PRKCA/IFNA1/PDGFA/FASLG/RAC1/MYB/RELA/COL1A1/PIK3CG/PDPK1/RAF1/KRAS/COL2A1/IL3/EIF4E/IFNB1/IL6R/CASP9/GSK3B/NGF/SPP1/MAP2K1/BCL2L1/COMP/CDK2/THBS1/HSP90AA1/PDGFB/IRS1/VWF/CHRM1/CHRM2/FGF2/FGF8/MTOR/HSP90AB1/ERBB2/KIT/CCND2/PDGFRA/MAPK3/F2R/BCL2L11/ITGB1/PCK2/FLT3/KITLG/IL7/IL4/NTF3/FGFR2/PDGFRB/ANGPT2/CSH1/YWHAZ/CSF3/YWHAB/HGF/JAK1/YWHAE/FLT4/FGFR4/MET/PPP2R1A/RET/PTK2/FLT1/PRKAA1/CSH2/TGFA/NRAS/CSF1/PDGFD/CDK6/YWHAG/PPP2CA/TLR2/SYK/FGF7/ITGB3/KDR/ITGA4/ITGA3/FGF18/ITGA2/VEGFC/LAMA4/LAMA1/FGF4/PIK3CB/EPOR/FGF10/FGF23/COL4A1/JAK2/OSM/RELN/TNC/AKT2/STK11/ITGAV/TSC2/COL4A3 |
| hsa05417 | Lipid and atherosclerosis | 124 | 1.14E-39 | MMP1/TP53/SOD2/MMP9/MMP3/IKBKG/TRAF6/TNF/IL6/TRAF3/CASP3/NFE2L2/CYCS/JUN/TAB2/IL1B/MAPK1/TNFSF10/IFNA2/IKBKB/STAT3/TLR4/BCL2/CXCL8/IL18/HSPA8/IRAK1/CASP6/IRF3/HSPA4/BAX/FOS/NOS3/PPARG/SRC/NFKB1/HRAS/AKT1/CHUK/TNFRSF1A/PIK3CA/CCL5/PRKCA/IFNA1/FASLG/RAC1/RELA/CASP8/MAPK14/PDPK1/CASP1/CD40/APOA1/KRAS/HSPA5/ABCA1/IFNB1/VCAM1/CASP9/ABCG1/GSK3B/MAPK8/HSPA1A/APOA4/APOB/CCL2/BCL2L1/NFKBIA/FAS/HSPA1B/HSP90AA1/CD36/APAF1/POU2F3/CYBB/MAP2K4/HSP90AB1/HSPD1/ICAM1/CASP7/MAPK10/MAPK3/CD14/CCL3/RXRB/MAP3K5/NLRP3/ITPR1/CALM1/LDLR/PTK2/DDIT3/RHOA/CYP2C9/PLCG1/RAP1B/NRAS/ERN1/NCF1/CYP1A1/PYCARD/LBP/XBP1/VLDLR/CXCL1/TLR2/CYP2A6/RAP1A/CDC42/HSPA1L/AGER/TNFRSF10A/CYP2C8/CD40LG/CAMK2A/PIK3CB/CYP2B6/JAK2/NOX1/CALM2/CALM3/LYN/CAMK2G/AKT2 |
| hsa04010 | MAPK signaling pathway | 114 | 4.78E-17 | TP53/IKBKG/TRAF6/MECOM/INS/TNF/CASP3/IGF1R/MAPT/BDNF/JUN/PRKCG/TAB2/IGF1/IL1B/EGFR/MYC/MAPK1/IGF2/NR4A1/IKBKB/SRF/TGFB1/NGFR/NTRK2/GDNF/HSPA8/IRAK1/PRKCB/FOS/VEGFA/NTRK1/NFKB1/HRAS/AKT1/EGF/CHUK/TNFRSF1A/FGFR1/INSR/PRKCA/PDGFA/FASLG/RAC1/RELA/MAPK14/IL1R1/RAF1/KRAS/MAPK8/NGF/HSPA1A/IL1A/MAP2K1/PAK1/FAS/TGFBR2/HSPA1B/MAX/PDGFB/FGF2/FGF8/PRKACA/MAP2K4/ERBB2/KIT/MAPK10/PDGFRA/MAPK3/CD14/MAP3K5/FLT3/KITLG/TGFB2/NTF3/TRADD/FGFR2/PDGFRB/ANGPT2/HGF/FLT4/FGFR4/MET/RET/CACNA1C/FLT1/DDIT3/RAP1B/TGFBR1/TGFA/CACNA1D/NRAS/CSF1/NF1/PDGFD/PLA2G4A/CACNA1H/RAP1A/CDC42/HSPA1L/FGF7/KDR/BRAF/FGF18/HSPB1/VEGFC/CACNA1I/FGF4/FLNA/FGF10/FGF23/MEF2C/AKT2/CACNA1G |
| hsa04020 | Calcium signaling pathway | 89 | 2.88E-11 | PRKCG/GRIN2A/EGFR/NOS2/NOS1/NTRK2/GDNF/PRKCB/NOS3/VEGFA/NTRK1/SLC8A1/EGF/GRIN1/P2RX2/MCOLN1/FGFR1/PRKCA/PDGFA/VDAC1/NGF/P2RX4/ADRB2/PDGFB/P2RX3/CHRM1/CHRM2/FGF2/FGF8/PRKACA/GRIN2B/ERBB2/PDGFRA/CXCR4/F2R/GRIN3A/FGFR2/CD38/ITPR1/PDGFRB/P2RX7/GNAQ/EDNRB/SLC25A4/DRD1/CALM1/HGF/NTRK3/MYLK/GRIN2D/FLT4/FGFR4/MET/RET/CACNA1C/FLT1/CHRNA7/PLCG1/GRIN3B/TGFA/CACNA1D/CAMK1D/ORAI1/PDGFD/ASPH/ATP2A2/PPIF/PTK2B/CACNA1H/FGF7/KDR/ADRA1A/FGF18/VEGFC/CACNA1I/CAMK2A/FGF4/RYR1/FGF10/FGF23/GRM1/ATP2B1/HRH1/CHRM3/CALM2/CALM3/ADRB1/CAMK2G/CACNA1G |
| hsa04668 | TNF signaling pathway | 61 | 9.71E-17 | TNFAIP3/MMP9/MMP3/IKBKG/TNF/IL6/CREB1/TRAF3/CASP3/MMP14/ADAM17/JUN/TAB2/IL1B/MAPK1/IKBKB/XIAP/FOS/NFKB1/IL18R1/AKT1/CHUK/TNFRSF1A/PIK3CA/CCL5/RELA/CASP8/MAPK14/IRF1/RIPK1/IFNB1/VCAM1/TNFRSF1B/MAPK8/MAP2K1/CCL2/NFKBIA/FAS/PTGS2/IL15/MAP2K4/CSF2/ICAM1/CASP7/MAPK10/MAPK3/MAP3K5/FADD/TRADD/SOCS3/CSF1/LIF/DNM1L/CXCL1/CFLAR/EDN1/VEGFC/PIK3CB/RIPK3/AKT2/LTA |
| hsa04064 | NF-kappa B signaling pathway | 50 | 2.27E-12 | PARP1/TNFAIP3/IKBKG/TRAF6/TNF/TRAF3/CSNK2B/TAB2/IL1B/CSNK2A1/IKBKB/TLR4/BCL2/XIAP/CXCL8/ATM/IRAK1/PRKCB/NFKB1/TNFSF11/CHUK/TNFRSF1A/RELA/IL1R1/RIGI/CD40/RIPK1/VCAM1/BCL2L1/NFKBIA/PTGS2/ICAM1/PLAU/CD14/TRADD/CXCL12/ZAP70/PLCG1/CCL4/LBP/CXCL1/BTK/SYK/CFLAR/MALT1/TNFSF14/CD40LG/LYN/PRKCQ/LTA |
| hsa04140 | Autophagy - animal | 50 | 0.000125462 | TRAF6/RPS27A/INS/UBC/IGF1R/SQSTM1/HIF1A/MAPK1/BCL2/CTSD/HRAS/AKT1/PIK3CA/PTEN/CTSB/PDPK1/RAF1/KRAS/MAPK8/MAP2K1/BCL2L1/IRS1/PRKCD/MTOR/PRKACA/HMGB1/MAPK10/MAPK3/OPTN/ITPR1/IRS2/UBB/BECN1/LAMP2/PRKAA1/NRAS/ERN1/PPP2CA/PIK3C3/CFLAR/DAPK1/WDFY3/ATG7/BNIP3/CTSL/PIK3CB/AKT2/STK11/TSC2/PRKCQ |
| hsa04152 | AMPK signaling pathway | 41 | 2.09E-05 | INS/CREB1/IGF1R/IGF1/CCND1/CCNA2/HNF4A/PPARG/LEP/AKT1/PIK3CA/INSR/PDPK1/CFTR/CD36/ADIPOR1/IRS1/MTOR/FOXO1/ADIPOR2/SIRT1/PCK2/FASN/ADIPOQ/IRS2/LIPE/HMGCR/PPP2R1A/ACACA/PRKAA1/SLC2A4/PPP2CA/SREBF1/ADRA1A/PIK3CB/FBP1/PPARGC1A/CPT1B/AKT2/STK11/TSC2 |
| hsa04150 | mTOR signaling pathway | 39 | 0.019493252 | INS/TNF/IGF1R/PRKCG/IGF1/MAPK1/IKBKB/CLIP1/PRKCB/HRAS/AKT1/CHUK/TNFRSF1A/PIK3CA/PTEN/INSR/PRKCA/PDPK1/RAF1/KRAS/EIF4E/GSK3B/MAP2K1/IRS1/MTOR/MAPK3/FNIP1/RHOA/PRKAA1/NRAS/BRAF/WNT3A/PIK3CB/WNT5A/DVL1/AKT2/STK11/TSC2/ATP6V1E1 |
| hsa04137 | Mitophagy - animal | 30 | 0.004708842 | SP1/TP53/RPS27A/ARIH1/PRKN/UBC/SQSTM1/HIF1A/JUN/CSNK2B/CSNK2A1/SRC/HRAS/RELA/KRAS/MAPK8/E2F1/BCL2L1/HUWE1/VCP/MAPK10/CITED2/OPTN/UBB/BECN1/OPA1/TOMM40/NRAS/SMURF2/BNIP3 |

Table S3 GO pathway of zinc-stroke-related genes.

| ID | Description | PValue | geneID | Count |
| --- | --- | --- | --- | --- |
| GO:0006979 | response to oxidative stress | 5.25E-69 | NOS3/APOE/APP/IL6/JAK2/HBB/MMP9/MAPT/TP53/PSEN1/ADIPOQ/H19/BMP7/MMP3/IL1A/EDN1/PTGS2/MMP2/SOD1/PARK7/GAS5/PRNP/MIR21/AKT1/HP/MPO/PTGS1/PDCD10/HMOX1/UCP2/SOD2/SNCA/MAPK3/HIF1A/MAPK1/GPX3/CASP3/CD36/PARP1/GSR/PRKN/ALOX5/SQSTM1/HGF/G6PD/GCH1/BCL2/ATP13A2/CRYAB/SIRT1/SRC/PPARGC1A/NFE2L2/XRCC1/MAPK8/FGF8/HBA1/FOS/EGFR/BECN1/GPX4/NR4A2/MIRLET7B/TXN/WRN/MIR34A/AIFM1/CAT/HSPA1A/NAGLU/COL1A1/MBL2/BNIP3/NCF1/EZH2/LRRK2/AQP1/ATP7A/DHFR/ATM/CYP1B1/DAPK1/EPAS1/MIR17/SLC1A1/STAT1/TXNIP/JUN/ERCC6/PEX10/HSPA1B/FUT8/GPX2/ABL1/MAP3K5/GPX1/PEX5/FOXO1/APOA4/ABCC1/PRDX1/ATP2A2/PAX2/MDM2/TAT/DUOX1/NDUFA12/LONP1/SLC25A24/GSTP1/UCP1/ALAD/PEX2/PEX14/PEX12/GCLM/PYCR1/KDM6B/FXN/PDGFRA/PENK/SP1/CHUK/TRPM2/GSS/KAT2B/ABCB11/RELA/MET/TXNRD2/PDGFD/ERN1/SIN3A/ERCC1/PNKP/FOXP1/PPP1R15B/HDAC6/AXL/SIRT2/FABP1/KLF2/CD38/RCAN1/SLC7A11/PAWR/PRDX5/MAP2K4/OGG1/MMP14/BTK/ARNT/HDAC2/SOD3/MYB/PCNA/SELENOP/CAPN2/MTF1/HMOX2/PRKD1/ZC3H12A/CDK1/HSF1/TNFAIP3/RIPK1/STK24/ANXA1/TRPA1/RIPK3/PPIF/PRKAA1/PRKCD/FYN/HAO1/STAT6/PTK2B/PRDX2 | 178 |
| GO:0001819 | positive regulation of cytokine production | 3.29E-44 | APP/IL6/JAK2/TNF/SERPINE1/IL10/IL1B/INS/PNP/AGT/NLRP3/PSEN1/ADIPOQ/H19/F3/LPL/TLR4/IL1A/IL4/LEP/PTGS2/TGFB1/SOD1/IL18/B2M/PARK7/ABCC8/CD40LG/MIR21/LTA/NOS2/APOA2/HMOX1/AGER/HMGB1/F2R/SMAD3/C3/CD14/PF4/HIF1A/MIR145/CSF2/IL17A/GATA6/CD36/IFNG/GATA4/TLR2/IL13/MBP/IL2/HGF/MEFV/MIR144/CCL3/STAT3/SIRT1/TWIST1/SRC/IL33/HRAS/IL1R1/IL6R/CYBB/CD34/MIF/GAPDH/CASP8/CLU/IFIH1/CASP1/CD40/MAPK14/SETD2/IDO1/ADAM17/HSPA1A/CD4/ATP6AP2/FADD/NLRP12/PIK3CG/C5/LRRK2/HSPD1/IL18R1/CYP1B1/POLR3B/THBS1/MMP12/TRAF6/HSPB1/MMP8/MIR17/DDIT3/STAT1/CARD9/NOX1/PANX1/ELANE/HSPA1B/ABL1/CD55/IRAK1/IRF5/RUNX1/BRCA1/XBP1/PYCARD/ITK/FOXP3/RORA/GATA3/KIT/RNF135/SAA1/DDX3X/TLR3/CHUK/NODAL/ZBTB20/IRF1/RELA/EGR1/SPTBN1/FOXP1/RAD21/PIBF1/IL27RA/SCAMP5/CD274/TRIM32/IRF3/TRIM65/LBP/OSM/HSP90AA1/RIGI/WNT3A/IL15/FLT4/TRAF3/CGAS/ISL1/HLA-A/BTK/PRKCQ/MALT1/ARNT/HDAC2/MIR182/ADCYAP1/NR1H4/STAT5B/MYB/XIAP/ORM2/IL7/SYK/WNT5A/PRKCZ/RIPK1/ANXA1/P2RX7/ZFPM1/NAIP | 167 |
| GO:0050727 | regulation of inflammatory response | 5.16E-53 | APOE/APP/IL6/LDLR/JAK2/TNF/SERPINE1/MMP9/APOA1/PROC/IL10/IL1B/INS/AGT/NLRP3/IGF1/ADIPOQ/PPARG/LPL/TLR4/MMP3/MIR146A/IL4/TNFRSF1A/PTGS2/TGFB1/SOD1/IL18/F12/PARK7/ESR1/ACE2/MIR21/LTA/GBA1/MIR223/AGER/SNCA/SMAD3/C3/MIR145/IL17A/IFNG/TLR2/PPARA/ALOX5/IL13/IL2/HGF/MEFV/MIR144/CCL3/STAT3/SRC/AHSG/NT5E/HLA-DRB1/IL33/MIR199A1/ADA/IL1R1/NFKB1/GHRL/CHRNA7/CASP1/MAPK14/IDO1/PLA2G2A/SLC39A8/GGT1/NCF1/FABP4/BCR/NLRP12/PIK3CG/EDNRB/LRRK2/NEAT1/DNASE1L3/ATM/CCL5/CYP19A1/MMP8/MIR221/NFKBIA/ELANE/GPX1/ABCC1/BAP1/IL2RA/TNFRSF1B/PYCARD/GSTP1/FOXP3/ALOX15/KLKB1/RORA/FURIN/GATA3/BRD4/VPS35/SOCS3/SAA1/TLR3/KARS1/KLF4/MIR128-1/PPARD/RELA/MIRLET7G/FOXP1/ASH1L/AHR/LGALS1/APCS/IRF3/TRIM65/S100A9/MIR141/NR1H3/LBP/PSMB4/OSM/ADAMTS12/TNC/MIR204/DNASE1/CD47/IL15/ACP5/TRADD/RB1/PGLYRP2/TNFAIP6/PLCG1/ISL1/CTSC/BTK/ARNT/S100A8/NR1H4/STAT5B/MIR488/REG3A/TNFSF11/XIAP/ETS1/FXR1/PTPN6/SYK/VAMP7/WNT5A/MIR222/PDCD4/TNFAIP3/TNIP1/MIR205/RIPK1/CEBPA/ANXA1/NAIP/PRKCD/FYN/LYN | 164 |
| GO:0010038 | response to metal ion | 2.98E-61 | FGA/ASS1/MMP9/CPS1/OTC/MAPT/TNNT2/ADAMTS13/TTN/IL1A/EDN1/VCAM1/PTGS2/TH/B2M/PARK7/PRNP/ABCC8/AKT1/CAV1/ATP7B/HMOX1/UCP2/SOD2/SNCA/MAPK3/HFE/CD14/HIF1A/MAPK1/DLG4/PTH/RYR1/CASP3/FGF23/PARP1/PRKN/TNFRSF11B/TF/CALM3/G6PD/BRAF/BCL2/ATP13A2/MECP2/CREB1/TSPO/CALM1/NFE2L2/CALR/BACE1/MAPK8/MAOB/CYBB/DNMT3A/FOS/SLC6A3/TFRC/KCNMA1/EGFR/BECN1/CASP8/SETD2/CAT/SLC39A8/CACNA1H/CYP1A2/SMPD1/BNIP3/NCF1/FABP4/LRRK2/SHH/AQP1/ATP7A/THBS1/BGLAP/TFAP2A/SLC1A1/CASP9/TXNIP/JUN/CYP1A1/MCOLN1/BMP6/TUBA1A/CASR/TERT/KMT2A/ANK3/MDM2/TAT/CALM2/D2HGDH/ALG2/LONP1/SLC25A24/ALOX15/GPHN/MEF2C/ALAD/KIT/FXN/PPP2CA/PENK/CACNA1G/GPI/CHUK/TRPM2/GSS/FBP1/GLRA1/SLC25A12/HAAO/SLC40A1/KCNK3/LIG4/SYT1/ERCC1/GDI1/GLRA2/LTA4H/GRIA1/TRPV6/SLC30A10/SLC11A2/BSG/SLC30A8/NCSTN/PDX1/HVCN1/NEDD4/OGG1/MEF2A/TFF1/S100A8/SOD3/P2RX4/MT2A/FECH/AOC1/CDH1/SLC30A1/PCNA/SLC30A3/MTF1/ACO1/WNT5A/CDK1/SUMO1/SERPINF1/HSF1/ATP5F1D/CEBPA/P2RX7/PPIF/PRKAA1/PTK2B/AQP3 | 159 |
| GO:0043410 | positive regulation of MAPK cascade | 3.03E-42 | APOE/APP/ITGB3/IL6/JAK2/TNF/FGA/IL1B/INS/IGF1/FGFR1/ICAM1/TLR4/IL1A/EDN1/CCL2/LEP/TGFB1/SOD1/RET/VEGFA/MIR21/ADRB2/EPO/KDR/CTNNB1/PDGFRB/PDCD10/AGER/HMGB1/TGFBR1/F2R/MAPK3/NOTCH1/XDH/FGF2/GDF15/NTRK2/CD36/FGF23/EGF/PDGFB/GATA4/THPO/IGF1R/RAF1/HGF/BRAF/CCL3/TGFB2/SRC/HLA-DRB1/HRAS/IGFBP3/FGF8/GHRL/MIF/GH1/GCG/FLT1/EGFR/NOX4/INSR/MIRLET7B/CHRNA7/CD40/CCL11/PLA2G2A/NRG1/CD4/MAP2K1/NCF1/BMP2/EZH2/PIK3CG/LRRK2/THBS1/MIR27A/NTF3/TRAF6/CCL5/BMP4/NRP1/LIF/MIR221/CDC42/CARD9/NOX1/CCR1/ELANE/NTRK1/SOX2/FGF10/ABL1/MAP3K5/EDN3/CCN2/GHR/CASR/IRAK1/FGF4/FGF18/FGFR2/PYCARD/GRM1/ALOX15/ROCK1/MEF2C/RAP1A/OPRM1/AR/KIT/PAK1/TRAF7/PDGFRA/PDGFA/RAP1B/TLR3/NODAL/NOTCH2/NMNAT1/PRKCA/PDGFD/ERN1/ADRA1A/MID1/IAPP/IL11/IGF2/CDH2/CFLAR/ERBB2/TGFA/OSM/SLC30A10/TP73/CCL4/FLT4/PTPN1/MAP2K4/FGFR4/CD44/ADCYAP1/FLT3/TNFSF11/XIAP/SPI1/SYK/ZC3H12A/WNT5A/MIR222/IGFBP4/PRKCZ/RIPK1/P2RX7/NAIP/NTRK3/PTK2B/PRDX2 | 159 |
| GO:0001666 | response to hypoxia | 4.56E-73 | MTHFR/ACE/TNF/PLAT/CBS/MALAT1/VHL/LMNA/TP53/ADIPOQ/PPARG/BMP7/PSEN2/IL1A/ITGA2/EDN1/MIR146A/VCAM1/CXCL12/LEP/PTGS2/TGFB1/SLC6A4/MMP2/TH/VEGFA/MIR21/CAV1/EPO/NOS1/LTA/NOS2/CXCR4/SMAD4/HMOX1/AGER/UCP2/SOD2/SLC2A1/MIR140/MTOR/SMAD3/NOTCH1/NF1/HIF1A/ANGPT2/MIR145/GATA6/RYR1/CASP3/TLR2/PPARA/MIR210/BCL2/MB/MPL/ADM/CRYAB/SIRT1/MECP2/TGFB2/TWIST1/SRC/NFE2L2/XRCC1/ADA/CYBB/DNMT3A/FOS/TFRC/KCNMA1/BECN1/SLC9A1/NR4A2/CREBBP/CHRNA7/DPP4/RBPJ/MIR34A/PLOD1/CAT/KCNJ11/ADAM17/HSD11B2/BNIP3/ITPR1/BMP2/AQP1/ATP7A/ATM/THBS1/EPAS1/LIF/MIR17/CASP9/CYP1A1/CHRNB2/APAF1/PIK3CB/SLC2A4/CASR/IRAK1/TERT/KCND2/FGFR2/MDM2/DDAH1/LONP1/RORA/HSPG2/VEGFC/ALAD/EP300/MYOCD/CITED2/MIR106B/PENK/PPARD/EGR1/MYC/USF1/ALAS2/KCNK3/RAD21/CFLAR/STUB1/PLAU/EGLN1/SIRT2/FABP1/PML/SLC11A2/CD38/REST/MMP14/ARNT/SOD3/TERC/MYB/P4HB/CAPN2/CA9/CLDN3/HMOX2/ENO1/HSF1/EGLN2/SUV39H1/PRKAA1/P2RX2/P2RX3/CCNA2/SRF/EGLN3/PTK2B/AQP3 | 156 |
| GO:1901342 | regulation of vasculature development | 2.23E-39 | NOS3/ITGB3/IL6/KRIT1/TNF/SERPINE1/NPPB/IL10/IL1B/SPARC/APOH/PPARG/F3/IL1A/MIR146A/CXCL8/LEP/VEGFA/ABCC8/MIR21/MIR125A/KDR/CTNNB1/MIR223/CXCR4/PDCD10/HMOX1/C3/NF1/PF4/HIF1A/ANGPT2/MIR145/FGF2/GATA6/TGFBR2/CLDN5/ITGB1/GATA4/ALOX5/MIR210/MIR143/WARS2/ADM/STAT3/SIRT1/MECP2/PRL/TGFB2/TWIST1/NFE2L2/MIR199A1/CYBB/GHRL/CD34/FLT1/MIRLET7B/CHRNA7/CD40/MIR34A/PIK3CG/AQP1/MIR34C/CYP1B1/THBS1/HSPB1/NRP1/MIR17/MIR221/STAT1/JAK1/JUP/TJP1/PIK3CB/ABL1/GLUL/MIR424/TERT/RUNX1/FGF18/CCR3/BRCA1/XBP1/WNK1/DDAH1/DCN/ROCK1/HSPG2/VEGFC/ANGPTL3/MIR193A/MIR106B/TLR3/SP1/NODAL/MIR200B/MIR199B/GATA2/KLF4/CXCR2/FASLG/PRKCA/RECK/MIRLET7G/ADAM10/ADAMTS1/MIR18A/ERBB2/EGLN1/KLF2/PML/PRKCB/SIRT6/PLCG1/ISL1/PDPK1/COL4A3/ETS1/PRKD1/SMAD1/GREM1/ZC3H12A/WNT5A/MIR222/SERPINF1/TNFAIP3/MIR205/HMGA2/HHEX/PTK2B | 130 |
| GO:0045765 | regulation of angiogenesis | 1.03E-38 | NOS3/ITGB3/IL6/KRIT1/TNF/SERPINE1/NPPB/IL10/IL1B/SPARC/APOH/PPARG/F3/IL1A/MIR146A/CXCL8/LEP/VEGFA/ABCC8/MIR21/MIR125A/KDR/CTNNB1/CXCR4/PDCD10/HMOX1/C3/NF1/PF4/HIF1A/ANGPT2/MIR145/FGF2/GATA6/TGFBR2/CLDN5/ITGB1/GATA4/ALOX5/MIR210/MIR143/WARS2/ADM/STAT3/SIRT1/MECP2/PRL/TGFB2/TWIST1/NFE2L2/MIR199A1/CYBB/GHRL/CD34/FLT1/MIRLET7B/CHRNA7/CD40/MIR34A/PIK3CG/AQP1/MIR34C/CYP1B1/THBS1/HSPB1/NRP1/MIR17/MIR221/STAT1/JAK1/JUP/TJP1/PIK3CB/ABL1/GLUL/MIR424/TERT/RUNX1/FGF18/CCR3/BRCA1/XBP1/WNK1/DDAH1/DCN/ROCK1/HSPG2/VEGFC/ANGPTL3/MIR193A/MIR106B/TLR3/SP1/NODAL/MIR200B/MIR199B/GATA2/KLF4/CXCR2/FASLG/PRKCA/RECK/MIRLET7G/ADAMTS1/MIR18A/ERBB2/EGLN1/KLF2/PML/PRKCB/SIRT6/PLCG1/ISL1/PDPK1/COL4A3/ETS1/PRKD1/SMAD1/GREM1/ZC3H12A/WNT5A/MIR222/SERPINF1/TNFAIP3/MIR205/HMGA2/HHEX/PTK2B | 128 |
| GO:0019216 | regulation of lipid metabolic process | 6.12E-37 | F2/APOE/RNF213/LDLR/TNF/APOA1/IL1B/MAPT/APOB/INS/ADIPOQ/PPARG/IL1A/LEP/PTGS2/TGFB1/EPHX2/SOD1/AKT1/CAV1/AVP/APOA2/PDGFRB/SERPINA3/SNCA/MTOR/C3/FGF2/IFNG/PDGFB/PPARA/IGF1R/NR3C1/ADM/SIRT1/TWIST1/CREB1/TSPO/PPARGC1A/PLA2G6/NFKB1/MIR98/FLT1/APOC3/CLCN2/UGT1A1/GAL/MLXIPL/BMP2/PIK3CG/ASAH1/BGLAP/AKT2/SIRT3/ZMPSTE24/ACADVL/CYP27B1/IRS1/BMP6/STAR/ATP1A1/APOA4/SREBF1/APOC2/HULC/HNF4A/KAT5/BRCA1/OGT/H6PD/RORA/ANGPTL3/PNPLA2/ARV1/KIT/SREBF2/PDGFRA/PDGFA/ABCG1/KAT2B/ZBTB20/PPARD/ABCB11/EGR1/EEF1A2/TBL1XR1/PIBF1/BCL11B/MBTPS2/ASXL3/STUB1/NR1H3/SORBS1/SIRT2/FABP1/MIR204/SIRT6/REST/MIR192/FGFR4/MTMR2/SCT/LACTB/IRS2/MIR182/FLT3/NR1H4/FABP5/STAT5B/SOX9/NR1H2/CAPN2/APOBEC1/PRKD1/CCN1/ALK/MBTPS1/SNAI1/ANXA1/ADIPOR1/NR5A1/MIR96/PRKAA1/PRKCD | 124 |
| GO:0000302 | response to reactive oxygen species | 3.54E-57 | NOS3/APOE/IL6/HBB/MMP9/MAPT/H19/BMP7/MMP3/IL1A/EDN1/MMP2/SOD1/PARK7/MIR21/AKT1/HP/MPO/PDCD10/HMOX1/UCP2/SOD2/MAPK3/HIF1A/MAPK1/CASP3/CD36/SQSTM1/HGF/GCH1/BCL2/CRYAB/SIRT1/SRC/NFE2L2/MAPK8/HBA1/FOS/EGFR/BECN1/MIRLET7B/TXN/MIR34A/AIFM1/CAT/COL1A1/BNIP3/NCF1/EZH2/LRRK2/AQP1/ATP7A/DHFR/ATM/CYP1B1/MIR17/STAT1/TXNIP/JUN/ERCC6/PEX10/ABL1/MAP3K5/GPX1/PEX5/APOA4/PRDX1/PAX2/MDM2/GSTP1/UCP1/PEX2/PEX14/PEX12/KDM6B/FXN/PDGFRA/CHUK/TRPM2/RELA/MET/TXNRD2/PDGFD/ERN1/FOXP1/PPP1R15B/HDAC6/AXL/FABP1/KLF2/PAWR/PRDX5/MAP2K4/BTK/HDAC2/SOD3/MYB/PCNA/CAPN2/CDK1/HSF1/TNFAIP3/RIPK1/STK24/ANXA1/TRPA1/RIPK3/PPIF/PRKAA1/PRKCD/FYN/STAT6/PTK2B/PRDX2 | 114 |
| GO:0043025 | neuronal cell body | 6.04E-27 | APOE/APP/KCNQ1/TNF/PMM2/ASS1/FLNA/MAPT/APOB/PSEN1/PSEN2/ENO2/S100B/TH/SOD1/RET/SNCA/CDK5/CACNA1C/CASP3/NDUFS7/IGF1R/MBP/MAP2/ATP13A2/MPL/CRYAB/TGFB2/SRC/BACE1/CYBB/CRH/CLCN2/SLC6A3/MME/INSR/CYP17A1/FMR1/TUBB3/GAL/CD40/RTN4R/NCF1/LRRK2/CALCA/ATP7A/BGLAP/SLC1A3/SLC1A1/GAP43/CDC42/GABBR1/GABRA2/SNAP25/NTRK1/GRIA2/KIF5A/SLC6A2/HCFC1/SST/HDAC1/ABL1/GHR/CASR/KCND2/TNFRSF1B/PYCARD/CAD/PDYN/SLC8A1/OPRM1/VPS35/NDN/GABRA5/UBB/PENK/ASCL1/SHANK2/TRPM2/TUBB4A/GLRA1/PPP2R1A/PAFAH1B1/GRIN3A/TXNRD2/NGFR/CNTN2/GDI1/ADNP/GRIN3B/WDFY3/ZC4H2/CDK5R1/HDAC6/TRPV1/ZPR1/SMN1/GRIA1/ITGA4/PTPRF/SIRT2/CALB1/HSP90AA1/PCSK2/C4A/MAP2K4/DVL1/SMN2/PICALM/ADCYAP1/HSP90AB1/ADCY10/P2RX4/CHRM2/GFRA1/PMM1/CAPN2/NEGR1/FXR1/WASHC5/SERPINF1/SRD5A2/NUMA1/PRKCZ/P2RX7/TOP1/PRKAA1/P2RX2/FYN/NEUROG1/BRD1/PTK2B | 132 |
| GO:0031983 | vesicle lumen | 3.39E-35 | F5/GLA/APP/TTR/SERPINE1/FGA/APOA1/VWF/INS/PNP/IGF1/SPARC/APOH/RAB27A/ALB/CTSA/TGFB1/VEGFA/B2M/HP/MPO/KNG1/ARG1/HMGB1/SERPINA3/SERPINA1/RETN/C3/TIMP1/POMC/PF4/MAPK1/PLG/VCP/EGF/PDGFB/FN1/GSN/ALOX5/TF/HGF/TGFB2/DBH/AHSG/ADA/BACE1/CXCL1/ITIH4/CTSD/DYNC1H1/NFKB1/VCL/GHRL/MIF/ATG7/GCG/EGFR/CLU/HSPA8/NEU1/MAPK14/CAT/PROS1/THBS1/GUSB/MMP8/AGA/CSTB/ELANE/TIMP3/TIMP2/APAF1/JUP/MAN2B1/TUBB/PYCARD/ANXA2/GSTP1/ROCK1/VEGFC/ALAD/PDGFA/PENK/DDX3X/GPI/FASLG/LTF/HUWE1/CANT1/CSNK2B/UNC13D/IGF2/S100A9/HSP90AA1/APRT/A2M/SLPI/ALDOA/CTSC/ALDOC/CCT2/S100A8/HSP90AB1/FABP5/AOC1/SELENOP/RNASE3/PTPN6/ORM2/FTL/CYB5R3/TUBB4B/CDA/PRKCD/ACLY | 115 |
| GO:0060205 | cytoplasmic vesicle lumen | 1.24E-34 | F5/GLA/APP/TTR/SERPINE1/FGA/APOA1/VWF/INS/PNP/IGF1/SPARC/APOH/RAB27A/ALB/CTSA/TGFB1/VEGFA/B2M/HP/MPO/KNG1/ARG1/HMGB1/SERPINA3/SERPINA1/RETN/C3/TIMP1/POMC/PF4/MAPK1/PLG/VCP/EGF/PDGFB/FN1/GSN/ALOX5/TF/HGF/TGFB2/DBH/AHSG/ADA/BACE1/CXCL1/ITIH4/CTSD/DYNC1H1/NFKB1/VCL/GHRL/MIF/ATG7/GCG/CLU/HSPA8/NEU1/MAPK14/CAT/PROS1/THBS1/GUSB/MMP8/AGA/CSTB/ELANE/TIMP3/TIMP2/APAF1/JUP/MAN2B1/TUBB/PYCARD/ANXA2/GSTP1/ROCK1/VEGFC/ALAD/PDGFA/PENK/DDX3X/GPI/FASLG/LTF/HUWE1/CANT1/CSNK2B/UNC13D/IGF2/S100A9/HSP90AA1/APRT/A2M/SLPI/ALDOA/CTSC/ALDOC/CCT2/S100A8/HSP90AB1/FABP5/AOC1/SELENOP/RNASE3/PTPN6/ORM2/FTL/CYB5R3/TUBB4B/CDA/PRKCD/ACLY | 114 |
| GO:0062023 | collagen-containing extracellular matrix | 9.60E-22 | F2/COL4A1/APOE/SERPINC1/FBN1/SERPINE1/FGA/MMP9/APOA1/VWF/AGT/ADIPOQ/SPARC/APOH/ICAM1/F3/BMP7/CXCL12/TGFB1/MMP2/F12/LOX/ELN/KNG1/SERPINA3/SERPINA1/TIMP1/PF4/COL3A1/ANGPT2/GDF15/PLG/ACHE/PDGFB/FN1/F9/LGALS3/TGFB2/AHSG/ANXA5/CALR/ITIH4/CTSD/GH1/APOC3/ADAMTS2/CLU/COL5A1/LAMA4/COL1A1/CTSB/SHH/THBS1/MMP8/CSTB/ELANE/TIMP3/FGF10/TIMP2/GPC3/CCN2/APOA4/COL18A1/LAMA1/FGFR2/DCN/ANXA2/CTSF/NID1/HSPG2/ANGPTL3/VTN/DLG1/COL2A1/CDH13/LGALS1/APCS/CDH2/ADAMTS1/S100A9/HPX/AMBP/HSP90AA1/TNC/ADAMTS10/NCAM1/A2M/AEBP1/SLPI/S100A10/CTSC/FLG/S100A8/SOD3/ACAN/IFNA2/CTSL/ADAMTS4/COMP/LGALS4/ADAMTS3/COL4A3/ORM2/IL7/CCN1/GREM1/WNT5A/APLP1/SERPINF1/TGM2/ANXA1/TGFBI | 112 |
| GO:0009897 | external side of plasma membrane | 6.40E-23 | ACE/F2/ITGB3/LDLR/TNF/F10/FGA/EPOR/GP1BA/THBD/ICAM1/F3/TLR4/ITGA2/VCAM1/CXCL12/ABCA1/B2M/PRNP/CD40LG/KDR/CXCR4/HFE/CD14/HLA-B/IL17A/TGFBR2/PLG/CD36/GSR/ITGB1/CTLA4/FAS/IL13/CCR5/ABCB1/ITGAM/MPL/NT5E/ANXA5/HLA-DRB1/CALR/ADA/IL1R1/IL6R/FGF8/CD34/TFRC/INSR/CHRNA7/CD40/RTN4R/ECE1/CD4/MBL2/CTSB/ATP6AP2/ITGB2/CD8A/SELL/THBS1/CCR1/GRIA2/CHRNB2/MAP3K5/CD79A/SLC2A4/GHR/PLAUR/CCR3/IL2RA/ABCG2/ITGAL/AQP4/KIT/PDGFRA/ABCG1/CXCR2/FOLR1/GLRA1/FASLG/IDE/IL27RA/CA4/CDH13/CD274/ANPEP/TRPV1/ITGA4/PLAU/NCAM1/CTSK/LCT/ITGA3/HLA-A/COLEC11/ABCC4/HLA-C/ENPEP/P4HB/GFRA1/MOG/CD9/CAPN2/ITGAV/P2RX7/MUC16 | 107 |
| GO:0098857 | membrane microdomain | 4.45E-33 | NOS3/APP/JAK2/KCNQ1/TNF/MAPT/PSEN1/ICAM1/PSEN2/TNFRSF1A/PTGS2/ABCA1/SLC6A4/PARK7/PRNP/ACE2/CAV1/KDR/NOS1/CTNNB1/LAMP2/SLC1A2/TGFBR1/F2R/SLC2A1/MAPK3/PRKAR1A/CD14/MAPK1/TGFBR2/CD36/DMD/ITGB1/TLR2/IGF1R/FAS/TFPI/ITGAM/SRC/BACE1/CTSD/SLC6A3/KCNMA1/EGFR/MME/SLC9A1/INSR/PRKACA/CHRNA7/DPP4/ADAM17/CBL/RTN4R/CD4/ITGB2/LRRK2/SHH/CD8A/PTCH1/VDAC1/KCNA3/SLC1A1/ATP5F1A/CHRNB2/ITLN1/IRS1/CD79A/ATP1A1/PODXL/SLC2A4/CD55/KCND2/TUBB/TNFRSF1B/NPHS2/ANXA2/ABCG2/FURIN/PPP2CA/EZR/FASLG/ZAP70/ADRA1A/DLG1/CDH13/CDH2/HDAC6/ORAI1/SMO/SORBS1/LIPE/PLPP3/CAVIN1/PIKFYVE/S100A10/BTK/CDH1/CAPN2/SMURF2/IKBKB/HAS2/TNFRSF10A/FYN/LYN | 104 |
| GO:0030666 | endocytic vesicle membrane | 3.65E-16 | NOS3/APOE/LDLR/HLA-DQB1/APOB/B2M/ACE2/ADRB2/CAV1/AVP/LAMP2/LRP1/HLA-B/DLG4/CD36/EGF/TLR2/TF/SCARB2/HLA-DRB1/CALR/CYBB/TFRC/EGFR/CFTR/CD4/ATP7A/PTCH1/VAMP2/GRIA2/MCOLN1/PIK3C3/MDM2/CLTC/GRIA3/UBB/HLA-DRA/CAMK2G/RPS27A/CAMK2A/SYT1/ATP6V0A1/HLA-DQA1/GRIA1/TGFA/SMO/WNT3A/IGF2R/PIKFYVE/HVCN1/TYRP1/HLA-A/HLA-C/CHRM2/UBC/CD9/VAMP7/PICK1/WNT5A/HBEGF/HLA-DRB5/LYN | 62 |
| GO:0034702 | monoatomic ion channel complex | 3.11E-08 | KCNQ1/GRIN2B/ABCC8/KCNJ5/CACNA1C/DLG4/RYR1/GRIN2A/CALM3/KCNH2/CALM1/CLCN2/KCNMA1/PRKACA/CHRNA7/GRIN1/KCNJ11/CFTR/CACNA1D/CACNA1H/GABRG2/KCNA3/GABRA2/GRIA2/KCNQ2/CHRNB2/CLCNKB/KCND2/ANO1/CALM2/GABRB3/CLCNKA/GABRA1/GRIN2D/GABRA5/GRIA3/CACNA1G/CACNA1I/HTR3A/GLRA1/GRIN3A/GABRA6/GABRB2/GABRA3/GLRB/GLRA2/GABRR1/KCNQ5/GRIN3B/KCNV2/GABRE/GABRQ/ORAI1/GRIA1/TRPV6/CLCN1/KCNJ16/TRPC6/KCNJ4/KCNQ4/PTPA/PTK2B | 62 |
| GO:1902911 | protein kinase complex | 1.25E-06 | TGFBR1/CDK5/PRKAR1A/TGFBR2/IGF1R/INSR/PRKACA/DAPK1/MED12/IRS1/MAP3K5/IKBKG/PRKDC/CDK6/CCND2/PYCARD/CCND1/CSNK2A1/CHUK/ERN1/CSNK2B/CDK5R1/PARD3/TRAF3/CCNY/STK11/CDKN1A/CDK2/PCNA/ZBTB7A/CDK1/IKBKB/PRKCZ/PRKAA1/CCNA2 | 35 |
| GO:0034361 | very-low-density lipoprotein particle | 7.58E-07 | APOE/APOA1/APOB/APOH/LPL/APOA2/APOC3/APOL1/APOA4/APOC2/VLDLR | 11 |
| GO:0140297 | DNA-binding transcription factor binding | 5.35E-45 | FLNA/WFS1/VHL/NLRP3/TP53/MAX/PPARG/PITX2/PARK7/ESR1/NKX2-5/ACTB/CTNNB1/SMAD4/HMGB1/MAPK3/HDAC9/SMAD3/SMAD2/HIF1A/GATA6/PARP1/SMARCA4/VDR/GATA4/PPARA/BCL2/CDKN2A/STAT3/SIRT1/TWIST1/SRC/CREB1/PPARGC1A/NFE2L2/CALR/TCF7L2/SKI/DNMT3A/CD34/FOS/NR4A2/PRDM16/GSK3B/TBX5/CREBBP/RBPJ/MAPK14/MLXIPL/CHD4/NSD1/SMARCB1/HDAC4/HSPB1/EPAS1/CTBP1/TAF1/DDIT3/STAT1/NFKBIA/GATA1/JUN/JMJD1C/MED12/HCFC1/JUP/HDAC1/HNF4A/KAT5/PRKDC/RBBP8/FOXP3/GATA3/MEF2C/EP300/YAP1/AR/HDAC8/KAT6A/POU1F1/MYOCD/CITED2/SMARCE1/ASCL1/SP1/BCOR/CIITA/KAT2B/NKX2-1/NOTCH2/GATA2/KLF4/YY1/PPARD/FBP1/RELA/EIF4E/NEUROD1/MYC/USF1/CSNK2B/SIN3A/XPC/ARID1A/FOXP1/KAT8/RAD21/MED25/ZNHIT3/AHR/TRIM32/NCOR2/SIRT2/TP73/PRKCB/REST/NR4A1/RUNX2/HDAC3/RB1/PRDM5/E2F1/ISL1/NR0B2/MEF2A/ARNT/GMNN/HDAC2/NR1I2/FLT3/NR1H4/STAT5A/STAT5B/KDM1A/FHL2/ZFPM2/SOX9/ID3/NR1H2/LEF1/PCNA/TBP/YWHAZ/ETS1/HMGA1/SPI1/ZBTB7A/RBFOX2/HSF1/GFI1B/CBX5/CEBPA/SRY/HMGA2/KDM4C/TAL1/LMO2/LDB1/ZFPM1/HIF1AN/SRF/RBX1/HHEX/SP3/EGR2 | 165 |
| GO:0061629 | RNA polymerase II-specific DNA-binding transcription factor binding | 1.08E-36 | TP53/PPARG/PITX2/PARK7/ESR1/NKX2-5/ACTB/CTNNB1/SMAD4/HMGB1/HDAC9/SMAD3/SMAD2/HIF1A/GATA6/PARP1/SMARCA4/VDR/GATA4/PPARA/CDKN2A/STAT3/SIRT1/SRC/CREB1/PPARGC1A/NFE2L2/CALR/TCF7L2/DNMT3A/CD34/FOS/NR4A2/GSK3B/TBX5/CREBBP/RBPJ/MAPK14/MLXIPL/CHD4/NSD1/SMARCB1/HDAC4/HSPB1/EPAS1/CTBP1/TAF1/DDIT3/STAT1/NFKBIA/GATA1/JUN/JMJD1C/MED12/HDAC1/HNF4A/PRKDC/RBBP8/FOXP3/GATA3/MEF2C/EP300/AR/POU1F1/MYOCD/CITED2/SMARCE1/SP1/NKX2-1/NOTCH2/GATA2/KLF4/FBP1/RELA/NEUROD1/CSNK2B/SIN3A/XPC/ARID1A/FOXP1/KAT8/MED25/ZNHIT3/AHR/TRIM32/NCOR2/TP73/PRKCB/REST/NR4A1/HDAC3/RB1/ISL1/NR0B2/MEF2A/ARNT/HDAC2/NR1I2/FLT3/NR1H4/STAT5B/KDM1A/ZFPM2/NR1H2/LEF1/PCNA/TBP/ETS1/HMGA1/SPI1/ZBTB7A/HSF1/GFI1B/CEBPA/HMGA2/KDM4C/TAL1/LMO2/LDB1/ZFPM1/HIF1AN/SRF/RBX1/SP3/EGR2 | 125 |
| GO:0001216 | DNA-binding transcription activator activity | 2.90E-14 | TP53/ESR1/NKX2-5/CDC5L/SMAD4/SMAD3/SMAD2/HIF1A/ETV4/GATA4/PPARA/NR3C1/STAT3/CREB1/NFE2L2/NFKB1/HNF1A/FOS/NR4A2/PRDM16/TBX5/RBPJ/MLXIPL/EPAS1/TFAP2A/FLI1/DDIT3/GATA1/JUN/SOX2/FOXO1/SREBF1/IRF5/RUNX1/HNF4A/NKRF/FOXP3/PLAGL1/GATA3/MEF2C/AR/NDN/POU1F1/PAX6/SP1/MYCN/HOXA1/THAP11/GATA2/KLF4/YY1/TCF4/IRF1/WT1/RELA/EGR1/NEUROD1/MYC/ETV6/RORB/ARX/BCL11B/SIX3/RREB1/KLF13/IRF3/GLIS3/NR1H3/ZIC3/TP73/NR4A1/RUNX2/VEZF1/E2F1/RXRB/ISL1/MECOM/MEF2A/ZNF143/NEUROG3/NR2C2/NR1I2/NR1H4/STAT5B/MEOX2/SOX9/MYB/PAX5/NR1H2/LEF1/MTF1/ZNF208/ETS1/SPI1/SMAD1/HSF1/GFI1B/BACH1/CEBPA/CASZ1/NR1I3/POU2F3/SRF/HHEX/KLF7/STAT6/PGR/EGR2 | 108 |
| GO:0001228 | DNA-binding transcription activator activity, RNA polymerase II-specific | 4.07E-14 | TP53/ESR1/NKX2-5/CDC5L/SMAD4/SMAD3/SMAD2/HIF1A/ETV4/GATA4/PPARA/NR3C1/STAT3/CREB1/NFE2L2/NFKB1/HNF1A/FOS/NR4A2/PRDM16/TBX5/RBPJ/MLXIPL/EPAS1/TFAP2A/FLI1/DDIT3/GATA1/JUN/SOX2/FOXO1/SREBF1/IRF5/RUNX1/HNF4A/NKRF/FOXP3/PLAGL1/GATA3/MEF2C/AR/NDN/POU1F1/PAX6/SP1/MYCN/HOXA1/THAP11/GATA2/KLF4/YY1/TCF4/IRF1/WT1/RELA/EGR1/NEUROD1/MYC/ETV6/RORB/ARX/BCL11B/SIX3/RREB1/KLF13/IRF3/GLIS3/NR1H3/ZIC3/TP73/NR4A1/RUNX2/VEZF1/RXRB/ISL1/MECOM/MEF2A/ZNF143/NEUROG3/NR2C2/NR1I2/NR1H4/STAT5B/MEOX2/SOX9/MYB/PAX5/NR1H2/LEF1/MTF1/ZNF208/ETS1/SPI1/SMAD1/HSF1/GFI1B/BACH1/CEBPA/CASZ1/NR1I3/POU2F3/SRF/HHEX/KLF7/STAT6/PGR/EGR2 | 107 |
| GO:0044389 | ubiquitin-like protein ligase binding | 6.84E-23 | CBS/WFS1/TP53/CTNNB1/CXCR4/TGFBR1/DNM1L/PRKAR1A/SMAD3/SMAD2/HIF1A/VCP/PARP1/PRKN/ABCB1/KCNH2/SQSTM1/BCL2/PPARGC1A/NFE2L2/CALR/LRPPRC/SKI/VCL/EGFR/BECN1/CASP8/CLU/HSPA8/PRKACA/GSK3B/CD40/HSPA1A/HSPD1/TRAF6/ACTG1/STAT1/NFKBIA/TXNIP/JUN/JAK1/HSPA5/HSPA1B/FOXO1/IKBKG/CUL3/MDM2/BRCA1/TUBB/TNFRSF1B/HERC2/PAX6/UBB/GPI/RELA/CUL2/RPS27A/NGFR/YWHAE/TNK2/MID1/HSPA1L/HDAC6/TRIM37/STUB1/HSP90AA1/RIGI/PML/ATXN3/RB1/TRAF3/ARIH1/CCT2/STAT2/HSP90AB1/TPI1/CDKN1A/POLR2A/UBC/POU5F1/RTN4/YWHAZ/UBE2L3/FHIT/CHEK2/APC/SMAD1/SUMO1/RPL5/KDM4A/RIPK1/HLTF/BAG6/RBX1/ELOB/DIO2/LYN/PTK2B/EGR2/HSPA9 | 100 |
| GO:0031625 | ubiquitin protein ligase binding | 6.91E-24 | CBS/WFS1/TP53/CTNNB1/CXCR4/TGFBR1/DNM1L/PRKAR1A/SMAD3/SMAD2/HIF1A/VCP/PARP1/PRKN/ABCB1/KCNH2/SQSTM1/BCL2/PPARGC1A/NFE2L2/CALR/LRPPRC/SKI/VCL/EGFR/BECN1/CASP8/CLU/HSPA8/PRKACA/GSK3B/CD40/HSPA1A/HSPD1/TRAF6/ACTG1/NFKBIA/TXNIP/JUN/JAK1/HSPA5/HSPA1B/FOXO1/IKBKG/CUL3/MDM2/BRCA1/TUBB/TNFRSF1B/HERC2/PAX6/UBB/GPI/RELA/CUL2/RPS27A/NGFR/YWHAE/TNK2/MID1/HSPA1L/HDAC6/TRIM37/STUB1/HSP90AA1/RIGI/PML/ATXN3/RB1/TRAF3/ARIH1/CCT2/HSP90AB1/TPI1/CDKN1A/POLR2A/UBC/POU5F1/RTN4/YWHAZ/UBE2L3/FHIT/CHEK2/APC/SMAD1/SUMO1/RPL5/KDM4A/RIPK1/HLTF/BAG6/RBX1/ELOB/DIO2/LYN/PTK2B/EGR2/HSPA9 | 98 |
| GO:0004175 | endopeptidase activity | 3.88E-15 | ACE/F2/F10/PLAT/MMP9/CPS1/PROC/PSEN1/ADAMTS13/F11/REN/F3/PSEN2/MMP3/MMP2/F12/ACE2/HP/PLG/CASP3/F9/SPG7/MMP1/BACE1/CTSD/ADAMTS2/MME/CASP8/DPP4/CASP1/ADAM17/ECE1/CTSB/SHH/MMP12/MMP8/CASP9/MMP13/ELANE/MMP7/ZMPSTE24/MIPEP/AFG3L2/ADAMTS7/LONP1/PMPCA/KLKB1/CTSF/MMP10/FURIN/PAPPA/PMPCB/C1S/GZMB/C1R/LTF/IDE/ECE2/USP7/USP9X/ADAM22/GPAA1/MBTPS2/ADAMTS18/ADAM10/ADAMTS1/KEL/MMEL1/CFLAR/PLAU/CASP7/USP20/ADAMTS12/PCSK2/MMP24/ADAMTS10/ADAMTS17/CFI/NCSTN/CTSK/MMP14/TLL1/CTSC/MALT1/PITRM1/CTSL/ADAMTS4/MMP25/CFB/CAPN2/ADAMTS3/HPR/MBTPS1/PCSK7/PHEX/CASP6 | 96 |
| GO:0005216 | monoatomic ion channel activity | 4.37E-10 | KCNQ1/PSEN1/SLC6A4/GRIN2B/ABCC8/KCNJ5/CACNA1C/RYR1/GRIN2A/KCNH2/CYBB/CLCN2/KCNMA1/CHRNA7/GRIN1/KCNJ11/CFTR/CACNA1D/CACNA1H/GABRG2/ITPR1/AQP1/VDAC1/TRPM7/KCNA3/APOL1/SLC1A1/ATP5F1A/GABRA2/NOX1/PANX1/SNAP25/GRIA2/MCOLN1/KCNQ2/CHRNB2/CLCNKB/KCND2/ANO1/KCNK9/KCNK18/GABRB3/CLCNKA/ANXA2/TRPM3/GABRA1/OPRM1/GRIN2D/GABRA5/GRIA3/CACNA1G/CACNA1I/TRPM2/HTR3A/GLRA1/GRIN3A/GABRA6/KCNK3/GABRB2/GABRA3/GLRB/GLRA2/ATP5PO/GABRR1/KCNQ5/CLCN4/GRIN3B/KCNH8/KCNV2/GABRE/GABRQ/ASIC1/TRPV1/ORAI1/GRIA1/TRPV6/CLCN1/HVCN1/KCNJ16/P2RX4/TRPC6/KCNJ4/KCNQ4/ITGAV/NCS1/ATP5F1D/P2RX7/TRPA1/P2RX2/P2RX3/ASIC3/PTK2B | 92 |
| GO:0033218 | amide binding | 3.89E-11 | APOE/CST3/LDLR/CPS1/APOA1/HLA-DQB1/PPARG/TLR4/ITGA2/ITM2B/B2M/PRNP/GRIN2B/ADRB2/AGER/LRP1/HLA-B/CD36/ACHE/GRIN2A/TLR2/IGF1R/ITGAM/CRYAB/TGFB2/HLA-DRB1/CALR/BACE1/BCHE/MME/INSR/CLU/CHRNA7/TUBB3/GRIN1/RTN4R/ECE1/ITGB2/EDNRB/DHFR/VDAC1/SLC19A1/PLA2G4A/SLC1A3/SLC1A1/NFKBIA/ACADVL/GRIA2/PC/PEX5/GHR/FOLH1/HSPG2/FURIN/OPRM1/GRIN2D/FASN/GRIA3/PPIG/BRAP/FOLR1/HLA-DRA/IDE/RELA/SLC40A1/NGFR/NUP214/ADNP/IAPP/ANPEP/HLA-DQA1/GRIA1/SOAT1/LBP/FKBP6/TYMS/PICALM/HLA-A/SLC7A8/HLA-C/HSP90AB1/ENPEP/MCHR1/GAD2/SLC7A9/SRD5A2/PNPLA3/PPIF/HLA-DRB5 | 89 |
| GO:0005126 | cytokine receptor binding | 1.30E-19 | ITGB3/IL6/JAK2/TNF/BDNF/IL10/IL1B/IL1A/CCL2/IL4/CXCL12/CXCL8/TGFB1/IL1RN/VEGFA/IL18/CD40LG/EPO/LTA/TGFBR1/CSF3/SMAD3/PF4/SMAD2/CSF2/TGFBR2/CASP3/NGF/IFNG/NES/IL13/IL2/CCL3/PRL/TGFB2/IL33/CXCL1/IL6R/MIF/GH1/CSF1/CASP8/CCL11/ADAM17/IFNB1/FADD/C5/KITLG/NTF3/TRAF6/CCL5/LIF/IL3/STAT1/JAK1/NTRK1/PYCARD/VEGFC/GATA3/TNFSF10/IL5/FASLG/TNFSF14/PIBF1/IL11/TRIM37/CFLAR/OSM/CCL4/IL15/TRADD/TRAF3/PLCG1/IFNA1/CSH1/IFNA2/CSH2/CNTF/TNFSF11/AMH/IL7/SYK/GREM1/RIPK1 | 84 |

Table S4 KEGG analysis of the key genes.

| Category | ID | Description | Count | PValue | Genes |
| --- | --- | --- | --- | --- | --- |
| KEGG_PATHWAY | hsa04210 | Apoptosis | 24 | 5.40E-26 | PARP1, ITPR1, CFLAR, RELA, NFKB1, ERN1, IKBKB, MAPK8, CASP8, CASP3, DDIT3, TNFSF10, BCL2, FAS, BAX, BIRC5, MAPK1, FADD, RAF1, CTSD, TP53, CTSB, BCL2L1, MAPK3 |
| KEGG_PATHWAY | hsa05200 | Pathways in cancer | 36 | 6.67E-26 | RB1, ITGB1, CDKN1A, HSP90AB1, HDAC1, PTEN, CXCR4, HIF1A, FOXO1, RELA, EGFR, IKBKB, MAPK8, CASP8, MYC, CASP3, ERBB2, MAPK1, FADD, RAC1, MAPK3, CDKN2A, DAPK1, ITGA3, MTOR, NFKB1, VEGFA, IFNG, BCL2, BAX, BIRC5, FAS, RAF1, TP53, BCL2L1, NFE2L2 |
| KEGG_PATHWAY | hsa04140 | Autophagy - animal | 25 | 3.07E-25 | BECN1, PTEN, ITPR1, HIF1A, STK11, MAPK8, LAMP2, MAPK1, ATG7, CTSD, MAPK3, CTSB, DAPK1, BNIP3, PRKCD, TSC2, CFLAR, MTOR, ERN1, BCL2, WDFY3, PIK3C3, RAF1, SQSTM1, BCL2L1 |
| KEGG_PATHWAY | hsa05212 | Pancreatic cancer | 18 | 2.47E-21 | RB1, CDKN1A, CDKN2A, EGFR, MTOR, RELA, NFKB1, VEGFA, IKBKB, MAPK8, ERBB2, BAX, MAPK1, RAC1, RAF1, TP53, BCL2L1, MAPK3 |
| KEGG_PATHWAY | hsa05167 | Kaposi sarcoma-associated herpesvirus infection | 23 | 8.71E-21 | RB1, BECN1, CDKN1A, ITPR1, HIF1A, MTOR, RELA, NFKB1, VEGFA, IKBKB, MAPK8, CASP8, MYC, CASP3, FAS, BAX, MAPK1, PIK3C3, FADD, RAC1, RAF1, TP53, MAPK3 |
| KEGG_PATHWAY | hsa05417 | Lipid and atherosclerosis | 23 | 7.50E-20 | HSPA8, HSP90AB1, HSPA5, ITPR1, RELA, NFKB1, ERN1, IKBKB, MAPK8, CASP8, CASP3, DDIT3, CASP1, TNFSF10, BCL2, FAS, BAX, MAPK1, RAC1, TP53, NFE2L2, BCL2L1, MAPK3 |
| KEGG_PATHWAY | hsa05163 | Human cytomegalovirus infection | 23 | 2.03E-19 | RB1, CDKN1A, CDKN2A, ITPR1, CXCR4, TSC2, EGFR, MTOR, RELA, NFKB1, VEGFA, IKBKB, CASP8, MYC, CASP3, FAS, BAX, MAPK1, FADD, RAC1, RAF1, TP53, MAPK3 |
| KEGG_PATHWAY | hsa05131 | Shigellosis | 22 | 3.36E-17 | ITGB1, BECN1, BNIP3, PRKCD, ITPR1, FOXO1, EGFR, MTOR, RELA, NFKB1, IKBKB, MAPK8, CASP1, BCL2, BAX, MAPK1, PIK3C3, RAC1, SQSTM1, TP53, BCL2L1, MAPK3 |
| KEGG_PATHWAY | hsa05215 | Prostate cancer | 16 | 2.44E-16 | RB1, CDKN1A, HSP90AB1, PTEN, FOXO1, EGFR, MTOR, RELA, NFKB1, IKBKB, ERBB2, BCL2, MAPK1, RAF1, TP53, MAPK3 |
| KEGG_PATHWAY | hsa05165 | Human papillomavirus infection | 23 | 9.48E-16 | RB1, ITGB1, CDKN1A, HDAC1, ITGA3, PTEN, TSC2, FOXO1, EGFR, MTOR, RELA, NFKB1, VEGFA, IKBKB, CASP8, CASP3, FAS, BAX, MAPK1, FADD, RAF1, TP53, MAPK3 |

Table S5 GO analysis of the key genes.

| ONTOLOGY | Term | Description | Count | PValue |
| --- | --- | --- | --- | --- |
| BP | GO:0006915 | apoptotic process | 29 | 7.57E-25 |
| BP | GO:0043066 | negative regulation of apoptotic process | 24 | 2.65E-20 |
| BP | GO:0043065 | positive regulation of apoptotic process | 20 | 9.65E-19 |
| BP | GO:0045944 | positive regulation of transcription by RNA polymerase II | 25 | 8.80E-13 |
| BP | GO:0071456 | cellular response to hypoxia | 12 | 1.65E-12 |
| BP | GO:0006974 | DNA damage response | 15 | 2.09E-12 |
| BP | GO:0051726 | regulation of cell cycle | 13 | 2.51E-11 |
| BP | GO:0042981 | regulation of apoptotic process | 13 | 2.64E-11 |
| BP | GO:0070301 | cellular response to hydrogen peroxide | 9 | 8.16E-11 |
| BP | GO:0008625 | extrinsic apoptotic signaling pathway via death domain receptors | 8 | 9.28E-11 |
| CC | GO:0005737 | cytoplasm | 56 | 2.33E-20 |
| CC | GO:0005829 | cytosol | 51 | 1.76E-16 |
| CC | GO:0032991 | protein-containing complex | 17 | 3.78E-10 |
| CC | GO:0005634 | nucleus | 44 | 2.69E-09 |
| CC | GO:0031264 | death-inducing signaling complex | 5 | 3.30E-09 |
| CC | GO:0005739 | mitochondrion | 23 | 7.07E-09 |
| CC | GO:0042470 | melanosome | 8 | 4.07E-08 |
| CC | GO:0048471 | perinuclear region of cytoplasm | 15 | 9.18E-08 |
| CC | GO:0005635 | nuclear envelope | 9 | 3.70E-07 |
| CC | GO:0031265 | CD95 death-inducing signaling complex | 4 | 6.18E-07 |
| MF | GO:0042802 | identical protein binding | 36 | 1.61E-19 |
| MF | GO:0019899 | enzyme binding | 20 | 1.43E-17 |
| MF | GO:0031625 | ubiquitin protein ligase binding | 18 | 2.75E-16 |
| MF | GO:0005515 | protein binding | 67 | 1.70E-10 |
| MF | GO:0019901 | protein kinase binding | 15 | 1.11E-09 |
| MF | GO:0042826 | histone deacetylase binding | 9 | 1.38E-08 |
| MF | GO:0044877 | protein-containing complex binding | 12 | 2.60E-08 |
| MF | GO:0046982 | protein heterodimerization activity | 12 | 7.67E-08 |
| MF | GO:0002020 | protease binding | 8 | 1.18E-07 |
| MF | GO:0140297 | DNA-binding transcription factor binding | 9 | 1.30E-07 |

Table S6 MCODE filtering result of key genes.

| BetweennessCentrality | ClosenessCentrality | Degree | MCODE::Score (1) | name | NeighborhoodConnectivity |
| --- | --- | --- | --- | --- | --- |
| 4.20E-04 | 0.55 | 14 | 9.487179487 | ITPR1 | 44.42857143 |
| 5.32E-05 | 0.528 | 9 | 6.533333333 | EIF4G1 | 48.77777778 |
| 9.69E-05 | 0.532258065 | 9 | 6.377777778 | SERPINA1 | 41.88888889 |
| 1.03E-04 | 0.528 | 8 | 5.785714286 | RAC1 | 44.375 |
| 0.001972561 | 0.528 | 10 | 4.761904762 | COL18A1 | 30.6 |
| 2.45E-05 | 0.519685039 | 6 | 4.761904762 | VAMP7 | 36.5 |
| 0.00224797 | 0.6875 | 36 | 23.90804598 | NFE2L2 | 42.58333333 |
| 0.016931679 | 0.776470588 | 47 | 23.79310345 | BECN1 | 37.74468085 |
| 0.012087397 | 0.795180723 | 49 | 23.49462366 | CASP8 | 37.46938776 |
| 0.014820221 | 0.75862069 | 45 | 23.38461538 | HSPA5 | 37.66666667 |
| 0.004862089 | 0.70212766 | 38 | 22.93596059 | FOXO1 | 40.55263158 |
| 0.009723595 | 0.76744186 | 46 | 22.90552585 | NFKB1 | 38.56521739 |
| 0.028696008 | 0.904109589 | 59 | 22.90552585 | TP53 | 34.6440678 |
| 0.024199387 | 0.88 | 57 | 22.90552585 | CASP3 | 35.38596491 |
| 0.009448839 | 0.76744186 | 46 | 22.90552585 | PARP1 | 38.5 |
| 0.019449519 | 0.825 | 52 | 22.90552585 | HIF1A | 36.61538462 |
| 0.014411117 | 0.795180723 | 49 | 22.90552585 | PTEN | 37.14285714 |
| 0.041234113 | 0.916666667 | 60 | 22.90552585 | GAPDH | 33.81666667 |
| 0.013490203 | 0.814814815 | 51 | 22.90552585 | BCL2L1 | 37.15686275 |
| 0.019539972 | 0.868421053 | 56 | 22.90552585 | BCL2 | 35.875 |
| 0.008669823 | 0.75 | 44 | 22.84137931 | SIRT1 | 38.70454545 |
| 0.010898131 | 0.733333333 | 42 | 22.35483871 | IFNG | 38.78571429 |
| 0.012945758 | 0.776470588 | 47 | 22.35483871 | MAPK3 | 37.61702128 |
| 0.005523527 | 0.6875 | 36 | 22.26984127 | CDKN2A | 40.55555556 |
| 0.039565404 | 0.825 | 52 | 22.09090909 | EGFR | 35.36538462 |
| 0.003130529 | 0.70212766 | 38 | 22.09090909 | RELA | 41.84210526 |
| 0.003022316 | 0.680412371 | 35 | 21.92364532 | CDKN1A | 41.51428571 |
| 0.007150802 | 0.725274725 | 41 | 21.90909091 | MAPK8 | 39.87804878 |
| 0.024455408 | 0.814814815 | 51 | 21.64705882 | HSP90AB1 | 35.70588235 |
| 0.003749348 | 0.680412371 | 35 | 21.39076923 | CASP1 | 40.37142857 |
| 0.003160194 | 0.673469388 | 34 | 20.95238095 | IKBKB | 41.82352941 |
| 0.004543715 | 0.680412371 | 35 | 20.80788177 | MAPK1 | 41.25714286 |
| 0.014668809 | 0.75 | 44 | 20.69950739 | ATG7 | 37.81818182 |
| 0.01297033 | 0.717391304 | 41 | 20.33103448 | SQSTM1 | 38.24390244 |
| 0.002166249 | 0.653465347 | 31 | 19.86324786 | FADD | 42.58064516 |
| 0.001005901 | 0.622641509 | 26 | 19.74025974 | CFLAR | 43.03846154 |
| 0.006463931 | 0.66 | 32 | 19.55555556 | HDAC6 | 41.96875 |
| 0.003537978 | 0.653465347 | 31 | 19.20289855 | TSC2 | 40.80645161 |
| 0.003060395 | 0.61682243 | 26 | 18.67099567 | ERN1 | 42.07692308 |
| 0.003128308 | 0.634615385 | 29 | 18.67099567 | CXCR4 | 41.86206897 |
| 0.001104698 | 0.611111111 | 24 | 18.58874459 | STK11 | 44.45833333 |
| 0.013800901 | 0.694736842 | 37 | 18.10507246 | HSPA8 | 36.89189189 |
| 0.004443341 | 0.640776699 | 29 | 17.9673913 | CTSB | 41.68965517 |
| 0.001176911 | 0.622641509 | 26 | 17.74285714 | BAX | 43 |
| 5.49E-04 | 0.605504587 | 23 | 17.57142857 | BIRC5 | 45.39130435 |
| 9.56E-04 | 0.605504587 | 24 | 17.45454545 | DDIT3 | 43.125 |
| 0.001618514 | 0.628571429 | 27 | 16.95652174 | RAF1 | 43.48148148 |
| 0.001376924 | 0.605504587 | 24 | 15.32631579 | FAS | 40.33333333 |
| 0.004849131 | 0.605504587 | 23 | 15.24210526 | CTSD | 40.65217391 |
| 0.001338554 | 0.578947368 | 19 | 14.77941176 | TNFSF10 | 43.10526316 |
| 0.001076909 | 0.611111111 | 24 | 14.60869565 | BNIP3 | 43.29166667 |
| 0.001899527 | 0.594594595 | 21 | 13.78571429 | LAMP2 | 41.42857143 |
| 0.004184708 | 0.578947368 | 19 | 13.76666667 | ITGB1 | 41.68421053 |
| 0.001379056 | 0.594594595 | 21 | 12.82105263 | PRKCD | 42.71428571 |
| 7.46E-04 | 0.578947368 | 18 | 12.33088235 | SIRT2 | 42.77777778 |
| 0.001323669 | 0.578947368 | 19 | 11.65714286 | TP73 | 40.52631579 |
| 8.69E-04 | 0.568965517 | 18 | 10.76470588 | DAPK1 | 40.33333333 |
| 0.036763394 | 0.88 | 57 | 23.4375 | MYC | 34.57894737 |
| 0.017302139 | 0.804878049 | 50 | 23.4375 | MTOR | 36.94 |
| 0.009865149 | 0.680412371 | 35 | 22.77 | HDAC1 | 40.2 |
| 0.01387069 | 0.717391304 | 40 | 21.56 | ERBB2 | 37.825 |
| 8.24E-04 | 0.589285714 | 21 | 16 | RB1 | 43.61904762 |
| 0.009146975 | 0.640776699 | 29 | 14.96 | PIK3C3 | 38.06896552 |
| 6.36E-05 | 0.536585366 | 11 | 10 | NAMPT | 44.90909091 |
| 0 | 0.474820144 | 5 | 5 | WDFY3 | 38.6 |
| 6.41E-04 | 0.528 | 8 | 5 | P4HB | 39.625 |
| 1.30E-04 | 0.488888889 | 5 | 4 | ITGA3 | 31.2 |

Table S7 Drug prediction results.

| gene | drug | drug_abbreviation | interaction score |
| --- | --- | --- | --- |
| NAMPT | GMX1777 | GMX1777 | 20.88151942 |
| NAMPT | DAPORINAD | DAPORINAD | 20.88151942 |
| TNFSF10 | RECOMBINANT ADENOVIRUS 5 ENCODING TUMOR NECROSIS FACTOR-RELATED APOPTOSIS-INDUCING LIGAND | RECOMBINANT | 17.40126619 |
| NAMPT | TEGLARINAD CHLORIDE | TEGLARINAD | 10.44075971 |
| NAMPT | CHS-828 | CHS-828 | 10.44075971 |
| FADD | FAS LIGAND | FAS | 8.700633093 |
| TNFSF10 | SODIUM BUTYRATE | SODIUM | 2.485895169 |
| NAMPT | NITRIC OXIDE | NITRIC | 2.088151942 |
| RELA | PYROCATECHOL VIOLET | PYROCATECHOL | 1.969954663 |
| RELA | MULBERROFURAN H | MULBERROFURAN | 1.969954663 |
| RELA | BROMOPYROGALLOL RED | **BPR** | 1.969954663 |
| RELA | CYNAROPICRIN | CYNAROPICRIN | 1.969954663 |
| TNFSF10 | TRICHOSTATIN A | TRICHOSTATIN | 1.740126619 |
| DDIT3 | 6-DIAZO-5-OXO-L-NORLEUCINE | DON | 1.242947585 |
| CFLAR | BAY-11-7085 | BAY-11-7085 | 1.087579137 |
| RELA | CHEMBL:CHEMBL1940084 | AR-12 | 0.984977331 |
| RELA | MORUSINOL | MORUSINOL | 0.984977331 |
| RELA | CUDRAFLAVONE B | CUDRAFLAVONE | 0.984977331 |
| RELA | DEHYDROXYMETHYLEPOXYQUINOMICIN | **DHMEQ** | 0.984977331 |
| CFLAR | FINASTERIDE | FINASTERIDE | 0.932210689 |
| CFLAR | IDRONOXIL | IDRONOXIL | 0.932210689 |
| DDIT3 | 1,4-BENZOQUINONE | **1,4-BQ** | 0.828631723 |
| DDIT3 | PHOTOSENSITIZING AGENT | PHOTOSENSITIZING | 0.828631723 |
| DDIT3 | TOPOISOMERASE INHIBITOR | TOPOISOMERASE | 0.828631723 |
| RELA | DIOSMETIN | DIOSMETIN | 0.656651554 |
| RELA | CHRYSOERIOL | CHRYSOERIOL | 0.656651554 |
| RELA | TAMARIXETIN | TAMARIXETIN | 0.656651554 |
| RELA | ANDROGRAPHOLIDE | ANDROGRAPHOLIDE | 0.656651554 |
| RELA | ISORHAMNETIN | ISORHAMNETIN | 0.656651554 |
| RELA | QUERCETAGETIN | QUERCETAGETIN | 0.656651554 |
| RELA | KAEMPFERIDE | KAEMPFERIDE | 0.656651554 |
| CFLAR | BICALUTAMIDE | BICALUTAMIDE | 0.652547482 |
| DDIT3 | RO 31-8220 | RO | 0.621473792 |
| DDIT3 | PHENETHYL ISOTHIOCYANATE | PHENETHYL | 0.621473792 |
| DDIT3 | RECOMBINANT NEUROTROPHIC FACTOR | RECOMBINANT | 0.497179034 |
| DDIT3 | PROTEIN KINASE INHIBITOR | PROTEIN | 0.497179034 |
| RELA | PARTHENOLIDE | PARTHENOLIDE | 0.492488666 |
| CFLAR | ALLOPURINOL | ALLOPURINOL | 0.435031655 |
| DDIT3 | MANNITOL | MANNITOL | 0.414315862 |
| RELA | SORBINIL | SORBINIL | 0.393990933 |
| DDIT3 | PENICILLAMINE | PENICILLAMINE | 0.355127881 |
| DDIT3 | MITOGEN-ACTIVATED PROTEIN KINASE INHIBITOR | MITOGEN-ACTIVATED | 0.355127881 |
| RELA | CHEMBL:CHEMBL8483 | Sorafenib | 0.328325777 |
| RELA | EDASALONEXENT | EDASALONEXENT | 0.328325777 |
| RELA | ARTESUNATE | ARTESUNATE | 0.328325777 |
| DDIT3 | GLUTAMINE | GLUTAMINE | 0.310736896 |
| CFLAR | CABOZANTINIB S-MALATE | CABOZANTINIB | 0.296612492 |
| CFLAR | NINTEDANIB ESYLATE | NINTEDANIB | 0.261018993 |
| RELA | CHEMBL:CHEMBL1405979 | BMS-582949 | 0.246244333 |
| DDIT3 | ALPHA-TOCOPHEROL | α-TOC | 0.22599047 |
| DDIT3 | PROTEIN SYNTHESIS INHIBITOR | PROTEIN | 0.207157931 |
| RELA | RUTIN | RUTIN | 0.196995466 |
| RELA | GOSSYPETIN | GOSSYPETIN | 0.196995466 |
| DDIT3 | PROTEASOME INHIBITOR | PROTEASOME | 0.186442138 |
| DDIT3 | FENRETINIDE | FENRETINIDE | 0.184140383 |
| RELA | ISOLIQUIRITIGENIN | ILG | 0.164162889 |
| RELA | PECTOLINARIGENIN | PECTOLINARIGENIN | 0.164162889 |
| RELA | VORICONAZOLE | VORICONAZOLE | 0.164162889 |
| RELA | PLUMBAGIN | PLUMBAGIN | 0.151534974 |
| DDIT3 | DEFEROXAMINE | DEFEROXAMINE | 0.146229128 |
| RELA | FRENTIZOLE | FRENTIZOLE | 0.140711047 |
| DDIT3 | RECOMBINANT INTERLEUKIN-1 | RECOMBINANT | 0.138105287 |
| RELA | ACACETIN | ACACETIN | 0.131330311 |
| DDIT3 | CELECOXIB | CELECOXIB | 0.129860195 |
| NFE2L2 | SUFORAPHANE ISOSELENOCYANATE | SUFORAPHANE | 0.124740259 |
| NFE2L2 | CYTOPROTECTIVE AGENT | CYTOPROTECTIVE | 0.124740259 |
| NFE2L2 | GENE TRANSDUCTION AGENT | GENE | 0.124740259 |
| NFE2L2 | LAGASCATRIOL | LAGASCATRIOL | 0.124740259 |
| NFE2L2 | CHEMBL:CHEMBL128729 | Zileuton | 0.124740259 |
| NFE2L2 | ANDALUSOL | ANDALUSOL | 0.124740259 |
| DDIT3 | HYDROGEN PEROXIDE | HYDROGEN | 0.124294758 |
| DDIT3 | OXIDOPAMINE | OXIDOPAMINE | 0.124294758 |
| DDIT3 | PLATINUM | PLATINUM | 0.124294758 |
| RELA | CHEMBL:CHEMBL260560 | Imatinib | 0.123122166 |
| RELA | CHEMBL:CHEMBL502774 | Erlotinib | 0.123122166 |
| RELA | CHEMBL:CHEMBL406557 | Gefitinib | 0.123122166 |
| DDIT3 | ANTIOXIDANT | ANTIOXIDANT | 0.11837596 |
| RELA | KAEMPHEROL | KAEMPHEROL | 0.109441926 |
| RELA | CHEMBL:CHEMBL85826 | Tariquidar | 0.109441926 |
| RELA | 4',7-DIMETHOXY-5-HYDROXYGENISTEIN | Genistein | 0.109441926 |
| RELA | 6,4'-DIMETHOXYFLAVONE | DMF | 0.109441926 |
